# Human-origin probiotic cocktail increases short-chain fatty acid production via modulation of mice and human gut microbiome

**DOI:** 10.1038/s41598-018-30114-4

**Published:** 2018-08-23

**Authors:** Ravinder Nagpal, Shaohua Wang, Shokouh Ahmadi, Joshua Hayes, Jason Gagliano, Sargurunathan Subashchandrabose, Dalane W. Kitzman, Thomas Becton, Russel Read, Hariom Yadav

**Affiliations:** 1 Department of Internal Medicine-Molecular Medicine, Wake Forest School of Medicine, Winston-Salem, NC USA; 2 Department of Microbiology and Immunology, Wake Forest School of Medicine, Winston-Salem, NC USA; 3 National Center for the Biotechnology Workforce, Forsyth Technical Community College, Winston-Salem, NC USA; 4 Department of Internal Medicine-Gerontology and Geriatric Medicine, Wake Forest School of Medicine, Winston-Salem, NC USA

## Abstract

The gut bacteria producing metabolites like short-chain fatty acids (SCFAs; e.g., acetate, propionate and butyrate), are frequently reduced in Patients with diabetes, obesity, autoimmune disorders, and cancers. Hence, microbiome modulators such as probiotics may be helpful in maintaining or even restoring normal gut microbiome composition to benefit host health. Herein, we developed a human-origin probiotic cocktail with the ability to modulate gut microbiota to increase native SCFA production. Following a robust protocol of isolation, characterization and safety validation of infant gut-origin *Lactobacillus* and *Enterococcus* strains with probiotic attributes (tolerance to simulated gastric and intestinal conditions, adherence to intestinal epithelial cells, absence of potential virulence genes, cell-surface hydrophobicity, and susceptibility to common antibiotics), we select 10 strains (5 from each genera) out of total 321 isolates. A single dose (oral gavage) as well as 5 consecutive doses of this 10-strain probiotic cocktail in mice modulates gut microbiome and increases SCFA production (particularly propionate and butyrate). Inoculation of these probiotics in human feces also increases SCFA production along with microbiome modulation. Results indicate that human-origin probiotic lactobacilli and enterococci could ameliorate gut microbiome dysbiosis and hence may prove to be a potential therapy for diseases involving reduced SCFAs production in the gut.

## Introduction

The human gastrointestinal tract harbors a highly diverse and complex community of 10^13^ to 10^14^ microorganisms (collectively known as the gut microbiome) living in a symbiotic manner^1^. This relationship is fundamental to host health and metabolism. Gut microbes facilitate host absorption and metabolism of complex nutrients through their wide array of enzymatic armory and biosynthetic capabilities^1,2^. Recent studies have demonstrated various adverse consequences of abnormal or altered gut microbiome (gut dysbiosis) including chronic gastrointestinal^3^, neurologic^4^, and metabolic^5^ diseases. Thus, the gut microbiome is receiving significant attention as a potential modifiable risk factor and therapeutic target^6^.

The mechanisms by which the gut microbiome affects host physiology may be at least partly mediated by short-chain fatty acids (SCFAs) which are the most abundant product of bacterial fermentation of undigested dietary fibers^7^. SCFAs can activate G-coupled protein receptors (GPCRs) including free fatty-acid receptor 2 and 3 (FFAR2/3)^8^, inhibit histone deacetylases^9,10^, and can be used as an energy substrate^11^, thereby positively affecting host’s physiological processes. Abnormally reduced intestinal levels of SCFAs or SCFA-producing gut bacteria are often found in patients with inflammatory bowel disease (IBD), obesity, type 2 diabetes, and autoimmune diseases^8,12–14^. This perturbation may be due to either diminished population of gut microbes that cross-feed SCFA-producers or an increased production of detrimental substances, either by host’s gastrointestinal tract or by other microbial cohabitants. Hence, developing and inoculating bacterial inhabitants (or co-inhabitants) that can cross-feed and stimulate/restore the population of SCFAs-producers in the gut could be a promising probiotic approach to help in the amelioration of various human diseases.

Several strategies including the use of antibiotics, prebiotics, and probiotics have been suggested to regulate the gut microbiome dysbiosis^15^. Probiotics are live microorganisms that, when administered in adequate amounts, confer a health benefit on the host^16^. Specific probiotic strains can effectively prevent or treat diseases including inflammatory bowel disease^17^, necrotizing enterocolitis^18^, obesity/type 2 diabetes^19^, and autoimmunity^20^ in both rodent models and humans; however, the mechanisms underlying such benefits are not understood. Nevertheless, these reports have led to an extensive demand for probiotic supplements over the last decade, thereby prompting a massive increase in the development of new probiotic products for the consumer market. However, these studies have mostly been conducted in animal models or human subjects with underlying pathologies, whereas reports on the effects of probiotics in healthy (disease-free) milieus remain relatively limited and inconsistent. Positive modulation of endogenous gut microbiome has been suggested as one of the mechanisms underlying probiotic effects^21,22^; however, whether (and how) probiotics affect the gut microbiome and SCFA spectrum in healthy hosts remains debatable^23^.

Lactic acid bacteria, which are extensively used as probiotics, can also promote the intrinsic growth of SCFA-producing gut bacteria^24,25^. To screen and select microbial strains with probiotic attributes, FAO and WHO have established some basic criteria^26^ such as tolerance to orogastrointestinal transit including high acid and bile concentrations, and adherence to human gastrointestinal mucosa. These traits ensure that probiotics survive gastric transit and reach the intestine in sufficient numbers to perform desired metabolic activities that are beneficial to the host. In addition, probiotic strains intended for human use must be of human origin and should be safe in terms of susceptibility to common antibiotics and absence of potential virulence factors and antibiotic-resistant genes. *Lactobacillus* and *Enterococcus* strains are found naturally in the human intestine^1^ and for this reason, such strains are frequently isolated from infants and adults so as to be tested and exploited as probiotics^27^. Multi-strain probiotic cocktails have been suggested to be effective against many intestinal illnesses^28,29^. Also, given that the human gut microbiome composition varies significantly according to the host’s geographical location^30,31^, probiotics isolated from local populations might be safer and more effective for native consumers. Probiotics are rapidly emerging as novel and natural therapeutic options; therefore, isolation and characterization of new strains with well-characterized mechanisms of action remain of considerable interest and importance. In this context, we herein develop a consortium of 10 probiotic *Lactobacillus* and *Enterococcus* strains isolated from healthy infants’ gut, and demonstrate their effects on the gut microbiome composition and SCFAs levels *in-vivo* in healthy mice as well as *ex-vivo* in human feces.

## Results

### Isolation and characterization of lactobacilli and enterococci from infant gut

**Figure 1:**
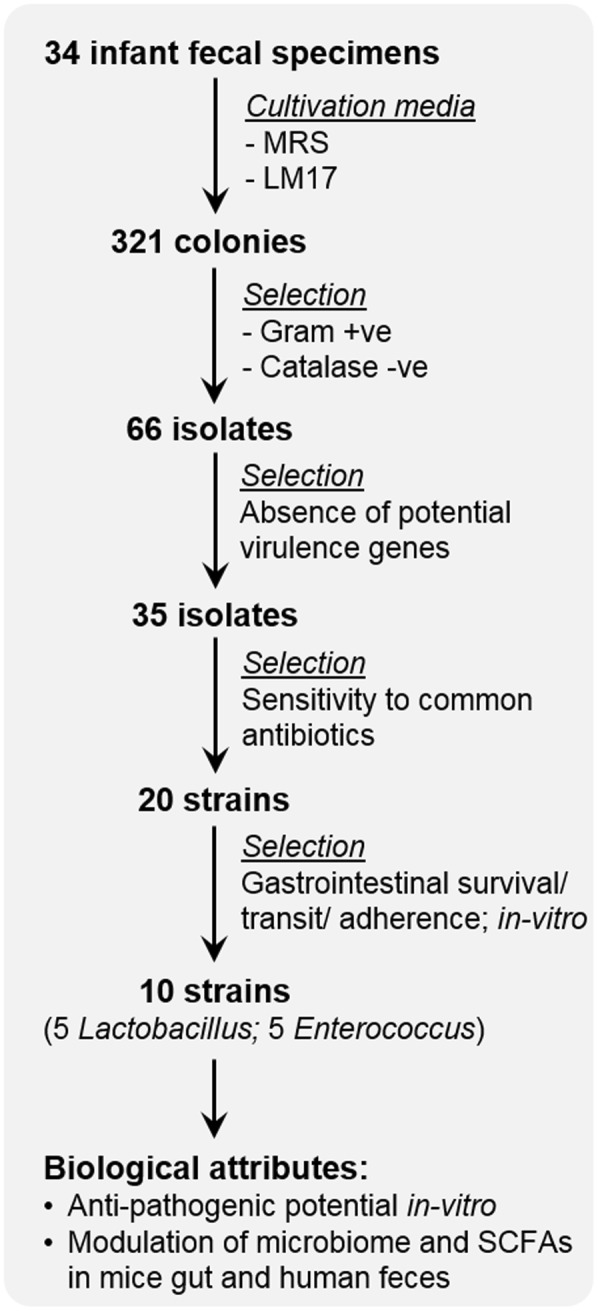
Flowchart depicting the process of isolation, characterization, and screening of human-origin probiotic lactobacilli and enterococci strains used in these experiments.

To isolate lactic-acid utilizing probiotic strains from 34 infant diapers, the fecal samples were cultured on MRS and M17 media supplemented with 35 mM lactic acid (LM17) (Fig. 1). A total of 321 colonies were selected; 66 colonies (34 from MRS and 32 from LM17) were Gram-positive and catalase-negative (Supplementary Table S1). After applying 16S rRNA gene sequencing and BLAST similarity search on selected MRS-grown isolates, 9 strains were identified as *Lactobacillus paracasei*, 20 strains as *L. rhamnosus*, 4 strains as *L. plantarum*, and 1 strain as *L. reuteri*. Among LM17-grown isolates, 5 strains belonged to *Enterococcus faecium*, 3 strains to *E*. INBio, 3 strains to *E. raffinosus*, 4 strains to *E. avium*, 10 strains to *E. faecalis*, 1 strain to *E. gallinarum*, 1 strain to *Streptococcus infantarius*, and 5 strains to *S. lutetiensis*. Further screening based on the absence of potential virulence genes (as detected by specific PCR assays) and the susceptibility to common antibiotics led to the short-listing of 16 isolates (9 lactobacilli and 7 enterococci; Supplementary Tables S2,S3 and S4) that were used for subsequent experiments.

### Probiotic properties of selected human-origin *Lactobacillus* and *Enterococcus* strains

In general, lactobacilli exhibited better probiotic attributes than *Enterococcus* strains (Table 1). Among *Lactobacillus* strains, *L. paracasei* D3-1, *L. paracasei* D3-2, *L. rhamnosus* D9-3, and *L rhamnosus* D12-6 were excluded based on weaker probiotic properties such as cumulative acid, bile, simulated gastric, and simulated intestinal tolerance, cell-surface hydrophobicity, and adherence to Caco2 cells. *L. paracasei D3-5* was selected over *L. casei* D3-1 and *L. paracasei* D3-2 to avoid the possibility of duplication of strains (these three colonies derived from the same infant) and also because it showed superior probiotic properties compared to the latter strains). *Enterococcus* strains showed weaker probiotic attributes than lactobacilli; however, we still short-listed selected *Enterococcus* strains to include enterococci in the probiotic consortium and to further study their effects in *ex-vivo* and *in-vivo* models. *E. gallinarum* D21-6 and *E. raffinosus* D25-3 were excluded from further studies due to their weaker probiotic attributes (Table 1). Overall, based on the arrays of different probiotic attributes, 5 strains of lactobacilli (*L. paracasei* D3-5, *L. rhamnosus* D4-4, *L. plantarum* D6-2, *L. rhamnosus* D7-5 and *L. plantarum* D13-4) and 5 strains of enterococci (*E. raffinosus* D24-1, *E*. INBio D24-2, *E. avium* D25-1, *E. avium* D25-2 and *E. avium* D26-1) were selected for further studies.

**Table 1:** Screening of *Lactobacillus* and *Enterococcus* strains for *in-vitro* probiotic attributes.

#	Isolates	Acid tolerance (%)	Bile tolerance (%)	Simulated gastric digestion tolerance (%)	Simulated intestinal fluid tolerance (%)	Surface hydrophobicity (%)	Adherence (%)
*1*	*L. paracasei* D3-1	99.9 ± 3.5	101.4 ± 1.0	97.3 ± 4.7	54.4 ± 6.5	14.2 ± 0.2	20.3 ± 4.8
2	*L. paracasei* D3-2	102.4 ± 5.3	103.3 ± 0.7	97.3 ± 5.3	54.6 ± 1.8	25.9 ± 0.2	20.4 ± 3.3
3	*L. paracasei* D3-5	102.9 ± 2.2	100.7 ± 1.4	100.1 ± 0.8	57.6 ± 2.5	29.6 ± 0.8	26.3 ± 3.2
4	*L. rhamnosus* D4-4	96.6 ± 3.0	101.5 ± 0.4	95.1 ± 3.5	54.9 ± 2.8	13.4 ± 0.0	14.7 ± 3.9
5	*L. plantarum* D6-2	101.9 ± 5.4	102.2 ± 1.6	102.9 ± 8.7	58.6 ± 3.8	10.5 ± 0.1	32.4 ± 3.6
6	*L. rhamnosus* D7-5	100.6 ± 1.7	101.8 ± 0.8	95.8 ± 0.7	54.2 ± 4.5	33.8 ± 0.3	15.5 ± 7.9
7	*L. rhamnosus* D9-3	98.0 ± 3.2	101.2 ± 2.4	97.2 ± 6.2	49.9 ± 4.6	11.2 ± 0.7	21.9 ± 9.8
8	*L. rhamnosus* D12-6	99.6 ± 5.3	94.5 ± 2.9	90.9 ± 10.7	50.8 ± 4.1	29.8 ± 1.4	17.4 ± 10.4
9	*L. plantarum* D13-4	101.7 ± 4.9	103.3 ± 2.0	98.9 ± 0.9	50.2 ± 6.5	6.3 ± 0.0	25.8 ± 3.8
10	*E. raffinosus* D24-1	64.0 ± 2.3	38.5 ± 0.3	0.4 ± 0.0	11.0 ± 0.7	13.6 ± 0.6	15.0 ± 0.4
11	*E*. INBio D24-2	72.4 ± 5.5	60 ± 0.5	0.4 ± 0.0	7.3 ± 0.5	46.9 ± 0.9	5.1 ± 0.1
12	*E. avium* D25-1	27.1 ± 1.2	72.5 ± 2.5	5.6 ± 0.4	13.2 ± 0.7	13.7 ± 0.7	9.0 ± 0.2
13	*E. avium* D25-2	6.3 ± 0.1	44.7 ± 0.3	8.4 ± 0.5	44.8 ± 0.1	15.6 ± 0.8	9.0 ± 0.5
14	*E. avium* D26-1	8.9 ± 0.1	58.4 ± 1.2	4.2 ± 0.2	25.5 ± 1.7	11.7 ± 0.8	44.2 ± 2.9
15	*E. gallinarum* D21-6	0.1 ± 0.0	86.4 ± 3.1	0.3 ± 0.0	84.7 ± 2.4	1.0 ± 0.4	1.0 ± 0.0
16	*E. raffinosus* D25-3	5.7 ± 0.4	63.2 ± 2.8	5 ± 0.6	63.0 ± 0.2	35.2 ± 0.5	13.7 ± 0.9

### Antimicrobial effects of probiotic lactobacilli and enterococci against uropathogenic *Escherichia coli* and *Klebsiella pneumonia*

**Figure 2:**
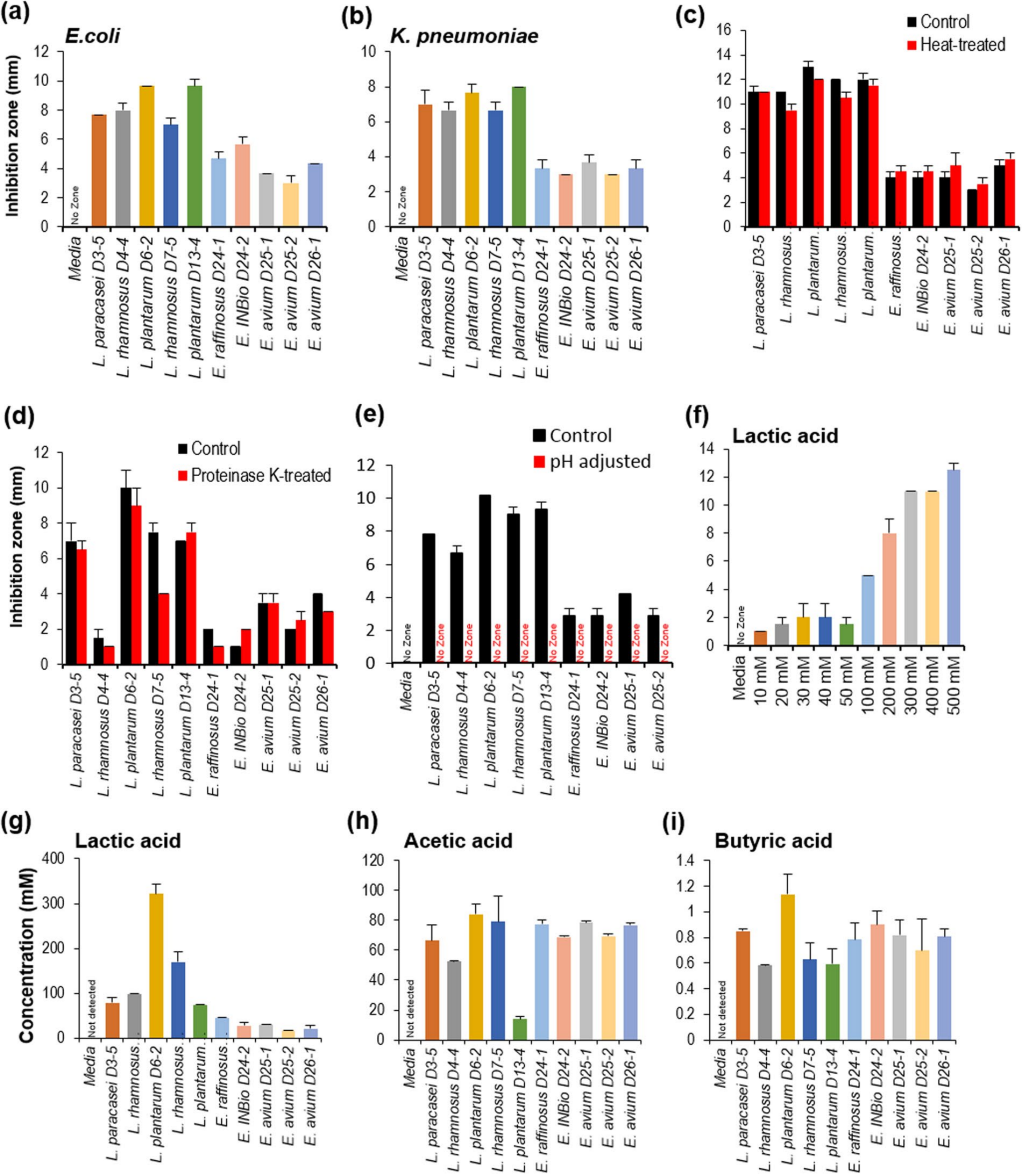
Human-origin probiotics inhibit uropathogenic bacterial growth. (**a**,**b**) Probiotic culture supernatant significantly reduced growth of clinical isolate of the human uropathogen *E. coli* (**a**) and *Klebsiella pneumoniae* (**b**) based on well diffusion assays. (**c**,**d**) Anti-microbial activity of probiotics supernatant against *E. coli* after heat (**c**) and proteinase K (**d**) treatments. (**e**) Neutralizing pH to 6.5 of probiotic supernatant abolished antimicrobial activity against *E*. *coli*. (**f**) Lactate inhibits *E. coli* in a dose-dependent fashion. (**g**–**i**) Probiotic produced significant amount of organic acid like lactate (**g**), acetate (**h**) and butyrate (**i**) in the supernatant. All the values plotted are means of 3–6 replicates or independent experiments and standard error of means. All the values are significantly different compared to media only (no-zone) control.

Selected probiotic strains (both lactobacilli and enterococci) inhibited the growth of clinical isolates of uropathogenic *E. coli* CFT073^32^ and *K. pneumoniae* KPPR1^33^ *in-vitro* (Fig. 2). Overall, lactobacilli strains exhibited a stronger antagonistic activity (i.e., a larger zone of inhibition) than enterococci strains against both pathogens in well-diffusion as well as agar-spot assays (Fig. 2a,b; Supplementary Fig. S1a–c). To further investigate if these antimicrobial effects were due to hydrogen peroxide produced by the probiotic strains, we measured the levels of hydrogen peroxide in the probiotic culture supernatants. We did not find detectable levels of hydrogen peroxide (Supplementary Fig. S2), indicating that the antimicrobial activities are independent of hydrogen peroxide. Moreover, since the heat treatment of probiotic culture supernatant exhibited no significant differences in their antimicrobial potential against uropahtogenic *E. coli* (Fig. 2c), antimicrobial components produced by these probiotics appear to be heat-stable. Proteinase K treatment of probiotics’ cell-free supernatants also did not affect their antimicrobial activity (Fig. 2d), suggesting that such activity is not due to protein mediators such as bacteriocins that are commonly produced by several probiotic strains^34,35^. However, neutralizing the pH of probiotics’ supernatant (to 6.5) diminished this antimicrobial activity (Fig. 2e), indicating that these probiotic strains exhibit antimicrobial activity against uropathogenic strains including *E. coli* and *K. pneumoniae* by producing higher levels of organic acids. This was further supported by the observation of profoundly higher levels of organic acids including lactic, acetic and butyric acids in probiotic culture supernatants (Fig. 2g–i). Lactic acid also showed potent growth inhibition against uropathogenic *E. coli* in a dose-dependent manner (Fig. 2f), thus suggesting that probiotic-derived organic acids inhibit the growth of uropathogens such as *E. coli* and *K. pneumoniae* in the gut.

### Impact of probiotic treatments on mouse gut microbiome and SCFAs level

To test the potential of selected human-origin probiotics to manipulate the gut microbiome and their capacity to produce SCFAs, we examined the effects of single dose as well as multiple doses of probiotics cocktail on gut microbiome composition and SCFAs levels in the mouse gut.

#### Single-dose study

**Figure 3:**
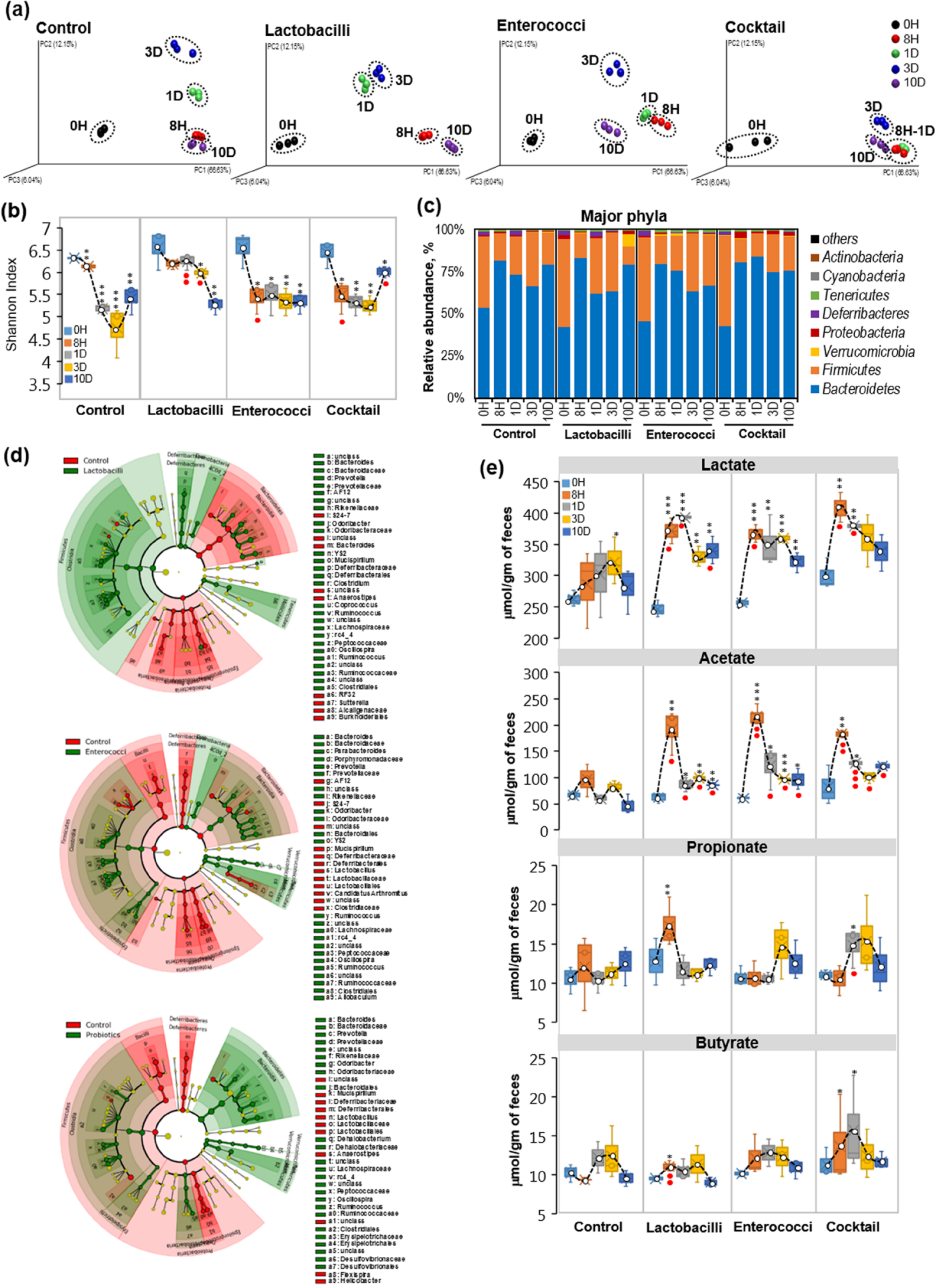
Single-dose feeding of human-origin probiotics causes mild changes in gut microbiome and increases SCFA production in the mouse gut. (**a**) PCA analysis showing the β-diversity clustering of gut microbiome from mice (n = 6 each group) treated with single-dose of five lactobacilli, five enterococci, and the cocktail containing all 10 strains, before treatment (0 h) and after 8 h, 1d, 3d and 10d of feeding. (**b**) Shannon index representing the alpha-diversity of gut microbiome at 0 h and after 8 h, 1d, 3d and 10d of lactobacilli, enterococci and cocktail treatments compared to non-treated control groups. (**c**) Major changes in bacteria phyla after a single dose of probiotics for 10 days. (**d**) Cladograms of linear discrimination analysis (LDA) showing clustering of gut microbiome after 1d of single-dose treatment of lactobacilli (upper panel), enterococci (middle panel) and cocktail (lower panel) compared to control group. (**e**) Fecal levels of SCFAs, lactate (upper panel), acetate (upper middle panel), propionate (lower middle panel) and butyrate (lower panel) increase after lactobacilli, enterococci and cocktail treatments. Values are mean ± SEM of n = 6 animals per group. Each dot in PCA analysis represents 2 samples pooled from 2 independent mice from same group, hence gut microbiome data will have this factor in all the data analyses at different time-points. SCFAs data represents duplicates of 5–6 and standard error of means. Values with ^*^<0.05, ^**^0.01 and ^***^0.001 are significantly different within the same group compared to 0 h time point. Values with ● < 0.05, ●● < 0.01 are significantly different compared to controls at the same time points.

Single-dose (oral gavage) of selected lactobacilli and enterococci probiotics to mice exhibited high variability in the β-diversity (Fig. 3a) of gut microbiome during different time-points. Alpha-diversity measures including Shannon Index (Fig. 3b), PD whole tree, Chao1 and observed OTUs (Supplementary Fig. S3a–d) were significantly decreased in all the groups, including control mice, after 8 h and days 1, 3 and 10 of treatment. However, compared to control group, the extent of decrease in α-diversity indices was less in groups treated with lactobacilli-alone, enterococci-alone, or the probiotic cocktail (lactobacilli plus enterococci) (Fig. 3b, Supplementary Fig. S3a–d).

The probiotic cocktail containing all 10 strains also significantly increased the abundance of *Bacteroidetes* while decreasing that of *Firmicutes* after day 1 of treatment (Fig. 3c,d, Supplementary Fig. S3d). However, lactobacilli-alone treatment slightly decreased *Bacteroidetes* and increased *Firmicutes*, while no such overall changes were observed in the enterococci-alone treated group (Fig. 3c; Supplementary Fig. S3e). Genus-level Lefse (linear discrimination analysis [LDA] effect size) analysis also showed that lactobacilli- and enterococci-alone treatments favored increased abundance of *Firmicutes* such as unclassified *Clostridia* while decreasing *Bacteroides*, *Prevotella* and *Ruminococcus* (Fig. 3d, Supplementary Fig. S4a–c; Supplementary Table S6). As expected, lactobacilli-alone treatment increased lactobacilli abundance after day 1. Enterococci-alone treatment significantly decreased lactobacilli groups while increasing *Verrucomicrobia* after day 1 of treatment (Fig. 3d, Supplementary Fig. 3f). Probiotic cocktail treatment decreased *Firmicutes* including lactobacilli and increased the abundance of *Bacteroidetes* (such as *Bacteroides* and *Prevotella*) and *Verrcomicrobia* at day 1 (Fig. 3d, Supplementary Figs S3g, S4a–c).

Probiotics (alone or in combination) significantly increased the levels of fecal lactate; the highest levels occurred after 8 h and/or day 1 of probiotic administration and then started decreasing after day 3. By day 10, levels of SCFAs were close to, but slightly higher than, baseline levels (Fig. 3e). Similarly, fecal acetate levels increased markedly in all treatment groups after 8 h and began returning toward baseline levels on day 1 and 3 post-treatment. They remained moderately higher up to 10 days post-treatment. Fecal propionate and butyrate levels increased significantly after 8 h and day 1 following the probiotic treatment but reverted to baseline levels by 10 days post-treatment (Fig. 3e). These results indicate that a single dose of human-origin probiotics may transiently modulate the gut microbiome composition and metabolic activity and enhance the production of intestinal SCFAs within 1–3 days.

#### Five-dose study

**Figure 4:**
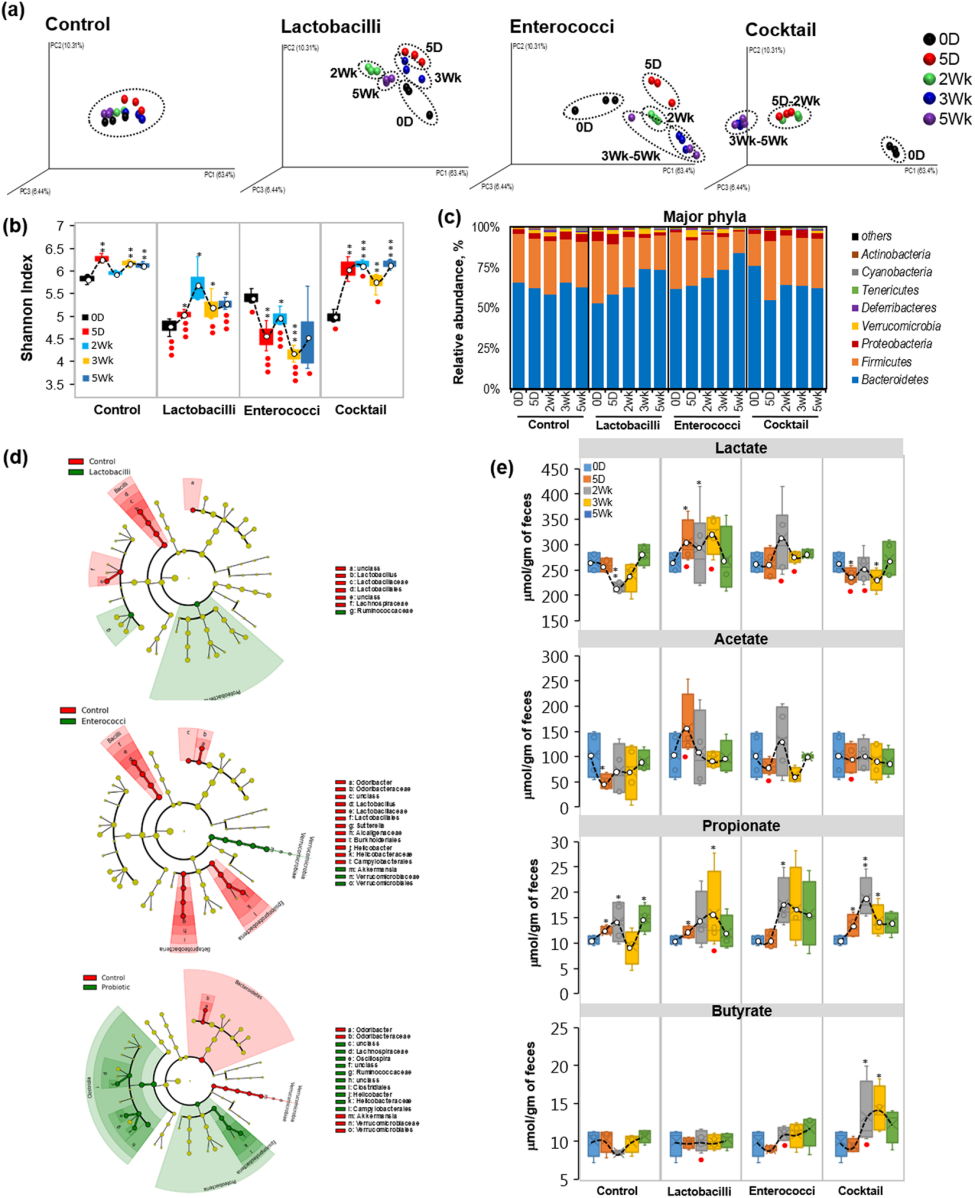
Five-day feeding of selected probiotics significantly increases microbiome diversity and SCFAs production in the mouse gut. (**a**) PCA analysis showing the β-diversity clustering of gut microbiome in mice after 5 days of once-daily feeding of lactobacilli, enterococci and their combination (cocktail) and non-treated control mice on 0d (before treatment), 5d, 2wks, 3wks and 5wks (after 1, 2 and 4 weeks of treatment). (**b**) Alpha-diversity (Shannon index) in mice undergoing 5 days of once-daily feeding of lactobacilli, enterococci and cocktail compared to control mice. (**c**) Major phyla changes upon 5 days of once-daily feeding of probiotic treatments up to 5 weeks. (**d**) Cladogram showing microbial signatures upon 5 days of once-daily feeding of lactobacilli, enterococci and cocktail compared to control mice. (**e**) SCFAs including lactate, acetate, propionate and butyrate levels after 5 days of once-daily feeding of probiotics treatment over the time course up to 30 days. Each dot in PCA analysis represents 2 samples pooled from 2 independent mice from same group, hence gut microbiome data will have this factor in all the data analyses at different time-points. SCFA data are duplicates of 5–6 and standard error of means. Values with ^*^<0.05, ^**^0.01 and ^***^0.001 are significantly different within the same group compared to 0 h time point. Values with ● < 0.05, ●● < 0.01 are significantly different compared to controls at the same time points.

We further investigated the impact of an extended regimen of probiotic feeding on murine gut microbiome and its metabolic function to produce SCFAs. We gave an oral gavage of the three probiotic combinations (same as those used in the single-dose study) once-daily for 5 consecutive days. Gut microbiome and fecal metabolites were monitored for up to 5 weeks post-treatment (Fig. 4). Feeding probiotics for 5 days induced significant changes in terms of β-diversity indices of the gut microbiome in probiotic cocktail-fed mice. With longer exposure, the clustering of the gut microbiome signature was notably different from that at the baseline within the same group (Fig. 4a). In addition, the lactobacilli- and enterococci-alone feeding also modulated the microbiome β-diversity clustering; however, these changes were more marked in enterococci-alone group compared to lactobacilli-alone fed mice, but much less compared than in the probiotic cocktail-treated group (Fig. 4a).

Alpha-diversity indices including PD whole tree, Chao1 and observed OTUs (Supplementary Fig S5a–c) showed high variability within the same group; however, Shannon Index (Fig. 4b) was relatively stable in the control group at all the time-points. Probiotic-cocktail and lactobacilli-alone treatments significantly increased, while enterococci-alone treatment decreased, the Shannon Index, indicating less microbial diversity. *Bacteroidetes* abundance were significantly increased in lactobacilli- and enterococci-alone treated groups, whereas levels of *Bacteroidetes* remained low in the probiotic cocktail-treated mice up to 5 weeks post-treatment, while Firmicutes and Proteobacteria remains in-consistently variable (Supplementary Figs S5d–g, S6). Again, enterococci-alone treatment appeared to favor an increase in the abundance of *Verrucomicrobia* after 5-dose treatments (Fig. 4c, Supplementary Figs S5h, S6b).

Lefse analysis showed that lactobacilli-alone feeding increased *Proteobacteria* and *Ruminococcaceae* while enterococci-alone feeding enhanced the abundance of *Verrucomicrobia* including *Akkermensia*. However, both treatments reduced the number of *Lactobacilliaceae* and *Lachnospiraceae* (Fig. 4d, Supplementary Fig. S6a–c, Supplementary Table S7). Probiotic-cocktail treatment significantly increased Clostridia, *Oscillospira*, *Lachinispiraceae* and *Ruminococcaceae* after day 5 (Fig. 4d). SCFAs analyses show that lactobacilli-alone treatment moderately increased the level of fecal lactate, while probiotic-cocktail treatment significantly decreased fecal lactate (Fig. 4e). No significant changes were observed in acetate levels in any of the treatment groups. Fecal propionate was significantly increased in all probiotic-treated groups, although this trend also was observed in the control group, thus indicating a high variability in propionate levels during the experiment independent of the treatments. Probiotic-cocktail treatment significantly increased fecal butyrate levels but not other organic acids after 2 weeks of treatment, although this increase tended to revert to baseline levels by the end of the study. This could indicate a microbiome modulation that eventually led to increased butyrate production in the gut.

### Impact of selected probiotics on human fecal microbiome

**Figure 5:**
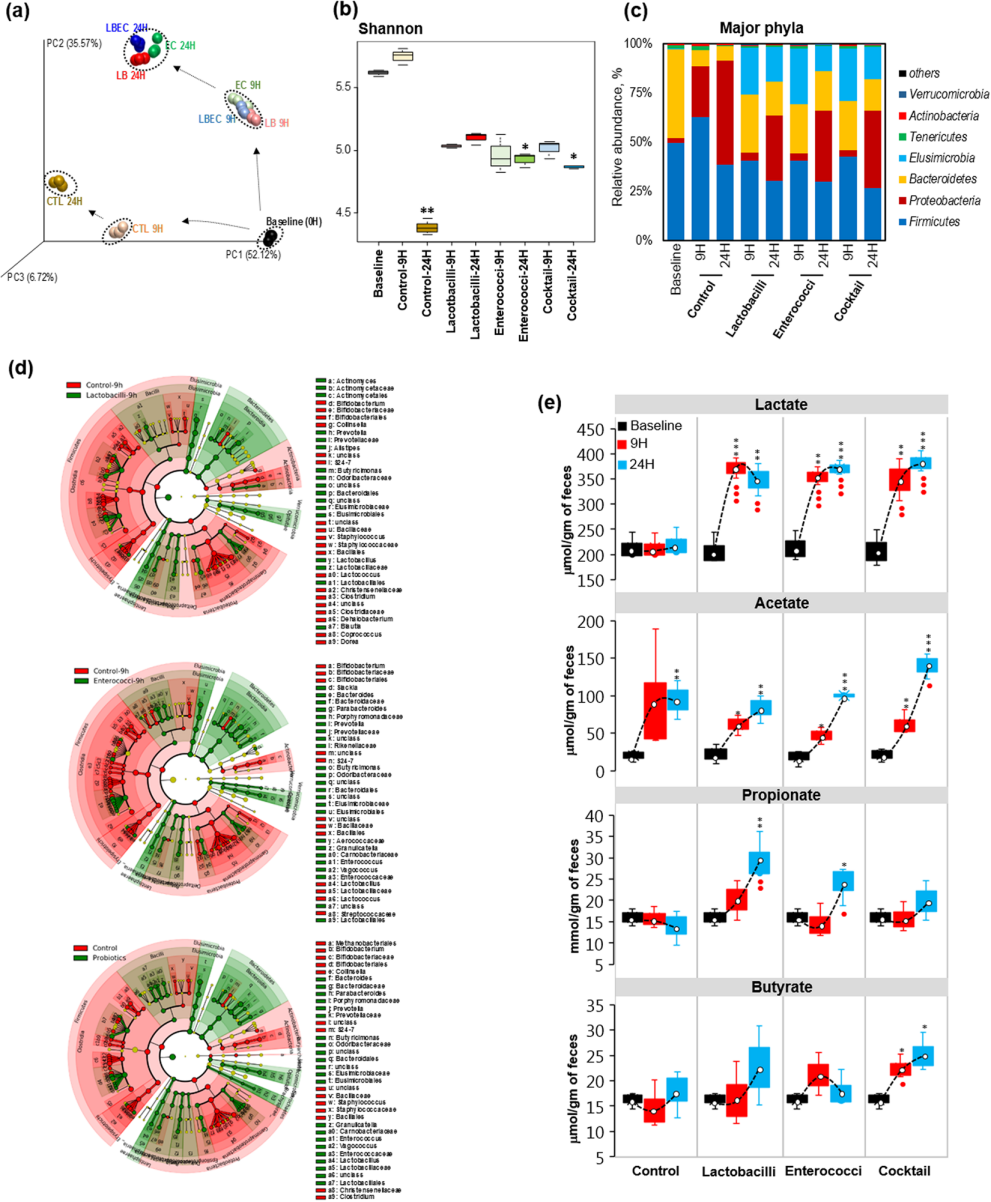
Probiotic inoculation differentially changes the human fecal microbiome ex-vivo with increased production of SCFAs. (**a**) PCA analysis of human fecal microbiome showing beta-diversity upon treatment with lactobacilli, enterococci and probiotics cocktail after 9 and 24 h of anaerobic incubation in conditioned medium following the probiotic inoculation. (**b**,**c**) Shannon index (**b**) and major phyla changes upon lactobacilli, enterococci and cocktail treatment over the time up to 24 h. (**d**) Microbial cladogram indicating microbial clustering of human fecal microbiome in lactobacilli, enterococci and cocktail treated specimen compared to control specimen. (**e**) SCFAs including lactate, acetate, propionate and butyrate levels in human fecal microbiome after lactobacilli, enterococci and cocktail treatment up to 24 h. Values with ^*^< 0.05, ^**^0.01 and ^***^0.001 are significantly different within the same group compared to 0 h time point. Values with ● < 0.05, ●● < 0.01 are significantly different compared to controls at the same time points.

To test the ability of selected probiotics to modulate the features of human gut microbiome and SCFAs spectrum, an *ex-vivo* human fecal slurry system was developed and employed to reflect and examine the features of human gut microbiome and intestinal organic environment. We inoculated the freshly-prepared human fecal suspension with probiotic combinations followed by incubation at 37 °C in an anaerobic chamber. Inoculation of lactobacilli- and enterococci-alone and the probiotic cocktail containing all 10 strains significantly modulated the β-diversity of gut microbiome, as reflected by noticeably distant clustering patterns compared with the control group. Alpha-diversity indices including PD whole tree, Chao1 and observed OTUs were significantly lower after 24 h in treated and non-treated samples (Supplementary Fig. S7a–d); however, Shannon Index was somewhat preserved in the treated samples, suggesting that the inoculation of probiotics helped in maintaining the microbial diversity in this fecal microbiome culture system. In control samples, the abundance of *Bacteroidetes* was significantly reduced while that of *Proteobacteria* was markedly increased over time (Fig. 5c). After 9 h incubation, the *Firmicutes* population was expanded in control samples while *Proteobacteria* abundance appeared to increase at the expense of *Bacteroidetes* and continued to expand further, thereby becoming a predominant population at 24 h of incubation. Probiotic-treated samples maintained a higher abundance of *Bacteroidetes* with no significant increase in *Firmicutes* up to 9 h; however, *Elusimicrobia* abundance appeared in all probiotic-treated samples at 9 h time. *Proteobacteria* were also increased in the probiotic-treated samples after 24 h of incubation whereas the abundance of *Firmicutes*, *Bacteroidetes*, and *Elusimicrobia*, tended to decrease. However, greater abundance of *Bacteroidetes* and lower abundance of *Firmicutes* were maintained after 24 h in probiotic-treated samples. Lefse analysis confirmed that the abundance of lactobacilli and enterococci was increased (Fig. 4d; Supplementary Fig. S8a–c), thus suggesting that the inoculated probiotics were able to survive/grow in the human fecal slurry. All probiotic treatments appeared to promote the growth of *Bacteroidetes* and *Prevotella*, *Butyricimonas*, *Bacteroides*, and *Elusimicrobiaceae* (Fig. 4d; Supplementary Fig. S8a–c; Supplementary Table S8). Further, lactate and acetate were significantly increased in all of the treatment groups (Fig. 4e). Propionate was significantly increased in lactobacilli- and enterococci-alone groups but not in the probiotic-cocktail group. As we saw in the mouse experiments, butyrate production was significantly increased in probiotic cocktail-treated samples but not in lactobacilli- and enterococci-alone treated samples (Fig. 4e). These results suggest that the selected human-origin probiotics could modulate human fecal microbiome diversity in a way enhancing the production of SCFAs in the gut.

## Discussion

Probiotic strains can help maintain and restore normal microbial balance (homeostasis) in the gastrointestinal tract by increasing the populations of beneficial bacteria while checking the growth of indigenous pathobionts and opportunistic pathogens^36^. An ideal probiotic strain intended for any human use must be (a) of human origin, (b) devoid of potentially virulence genes, (c) sensitive to common antibiotics, (d) tolerant to gastric and intestinal physico-chemical conditions; and should preferably be (e) catalase-negative, (f) able to adhere to intestinal epithelial membrane and (g) be able to compete with other microbes in the gut. Most of the commonly used probiotics (except *E. coli* Nissle 1917) are Gram-positive bacteria^37^, reasonably because these lack lipopolysaccharides (LPS), one of the most common pro-inflammatory components present in high proportion exclusively in Gram-negative bacteria^38^. Catalase-positive bacteria catabolize hydrogen peroxide into oxygen and water and hence can create a more oxygenated environment and reduce the anaerobic capacity of the gut microbial ecosystem or food systems. Thus, catalase-positive bacteria are generally not considered a good choice for use as probiotics. Our thorough screening for these characteristics successfully allowed us to select top 5 *Lactobacillus* and 5 *Enterococcus* strains possessing these desirable traits. We postulated that these selected probiotics would be better able to transit normal barriers in the gastrointestinal tract and survive in the lower gut.

Once probiotics reach to the lower part of the intestine, it must withstand and/or compete with the existing complex microbiota. Hence, an ability to produce antimicrobial substances may enhance the chances of a probiotic strain to successfully colonize the gut and overcome the microbial competition by preventing enteropathogen attachment and proliferation^34,36^. The final 10 probiotic isolates (5 *Lactobacillus* and 5 *Enterococcus* strains) short-listed in the present study exhibited significant antimicrobial properties against uropathogenic strains of *E. coli* and *Klebsiella pneumoniae* (Fig. 2). To better understand the mechanism(s) underlying this antimicrobial activity of probiotics, we tested four possible mechanisms: hydrogen peroxide production, heat-labile versus heat-stable factors, protein versus non-protein factors, and pH- or organic acid-mediated factors. Higher production of organic acids (i.e., lactate, acetate, and butyrate) appeared to be one of the major mechanisms driving these antimicrobial properties^39^. However, it would be interesting for further studies to test the potential of these (or such) probiotic isolates against uropathogens colonizing the gut and in urinary tract infections.

Bacterial fermentation in mouse cecum and human colon leads to the formation of SCFAs (lactate, acetate, propionate, and butyrate)^7^. SCFAs are critical for metabolism of epithelial cells; for example, butyrate is one of the major energy sources for enterocytes and colonocytes^40^. Blocking β-oxidation inhibits butyrate consumption in colonocytes, which ultimately results in ulcerative colitis due to deficiency in butyrate utilization in ileal and colonic mucosa^41^. SCFAs are also involved in the prevention of diarrhea (water and Na+ absorption)^42^. Decreased abundance of SCFAs is associated with increased leakiness of gut that allows diffusion of gut microbial contents into the circulation and leads to inflammation^43^. Butyrate increases expression of tight-junction proteins to reduce gut hyperpermeability that results in decreased inflammation/ endotoxemia associated with leaky gut^44^. Emerging literature suggests that gut microbiome-derived SCFAs may also modulate several important factors in host health such as immune cells^13^, hepatocytes^45^, muscles^45^, pancreatic cells^46^, adipose tissue^45^ and neuronal cells^47^. Most of these cells express SCFA receptors called FFAR2/3. This, along with the evidence that SCFAs are also found in the blood circulation, suggests that SCFAs can directly manipulate such cells to regulate host health^7,8,11,14,40^. Therefore, adequate production of SCFAs by microbiota in the gut is critical to maintain normal gut physiology and metabolic functions of the host. Hence, in the present study, we chose human-origin probiotics that can also enhance the capacity of gut microbiome to produce higher levels of SCFAs, with the ultimate goal of benefiting host health.

A more diverse gut microbiome is better able to produce higher levels of SCFAs^7^. Studying the complex microbial community inhabiting human colon presents some methodologic challenges. However, colonic models such as fecal slurry are seen as useful tools for *in-vitro* investigation of the composition and metabolism of colonic bacteria. Particularly, to obtain preliminary data about the effects of novel therapies including probiotics and prebiotics, such *in-vitro* (or *ex-vivo*) platforms can be very useful before an *in-humana* evaluation. Thus, we herein used mice as *in-vivo* models and human fecal slurry as *ex-vivo* models to test the potential of newly isolated human-origin probiotics to enhance gut microbial diversity and SCFA production.

Both single- and five-dose treatments caused limited changes in the microbial diversity (α- and β-diversity indices) but significantly increased SCFAs levels in the mouse gut (Figs 3 and 4). Probiotic treatment also significantly enhanced microbial diversity and increased SCFA production in human fecal microbiome (Fig. 5). Feeding of probiotics (mostly single strain) induces minimal microbiome changes but can still enhance microbial diversity to produce beneficial metabolites i.e., SCFAs in the gut^48^. Our results show that a probiotic cocktail containing multiple strains can cause a considerable shift in the gut microbiome signature, with increased SCFAs production. Abnormally high *Firmicutes* and low *Bacteroidetes* abundances are associated with imbalanced microbiome composition (gut dysbiosis) and several dysbiosis-associated diseases including diabetes, obesity, cancer and irritable bowel disease^49^. Although lactobacilli and enterococci both belong to *Firmicutes*, inoculation of these probiotics overall decreased *Firmicutes* and increased *Bacteroidetes* abundances in mice and human fecal microbiome (Figs 3–5). These findings suggest that treatment with such human origin-probiotics may help ameliorate gut microbiome dysbiosis by enhancing microbial diversity, *Bacteroidetes* abundance, and SCFAs production, while suppressing *Firmicutes* population.

Diverse microbes in the gut possess ability to produce SCFAs. Among these, *Bacteroides*, *Ruminococcaceae*, *Lachnospiraceae*, Clostridia, *Prevotella*, *Oscillospira* and *Verrucomicrobia* (*Akkermansia muciniphila*) are most commonly associated with increased production of SCFAs, especially propionate and butyrate^12,50,51^, possibly by cross-feeding each other. Our results also show that probiotic treatment enhanced *Bacteroides*, *Ruminococcaceae*, *Lachnospiraceae*, Clostridia, *Prevotella*, *Oscillospira*, and *Verrucomicrobia* (*Akkermansia muciniphila*) in mouse and human fecal microbiome, further suggesting that human-origin probiotic cocktails may enhance the abundance of butyrate producers. Overall, enhanced microbial diversity, decreased *Firmicutes*, and increased *Bacteroidetes* and SCFAs levels evidence that these (or such) probiotic *Lactobacillus* and *Enterococcus* strains (particularly, in combination) may be beneficial in ameliorating gut microbiome dysbiosis in several human diseases including obesity, type 2 diabetes, cancer, and IBDs, and might serve as potential biotherapy to prevent/cure such ailments.

Our study has some limitations. For instance, we did not test the influence of these probiotics in any disease phenotype. However, very few studies have examined the effects of probiotics on a healthy (disease-free) gut microbiome, which was our focus here. This also explains why we did not use any reference probiotic strains, since we sought to test how inoculation with probiotic-like strains could influence the diversity and composition of the gut microbiome under normal conditions. We only tested the effects of a single dose or five doses of probiotics; nonetheless, positive effects were observed even in these short-term regimens. Bifidobacteria and lactobacilli have been the most studied probiotic genera for various health benefits but bifidobacteria are already known to modulate gut microbiome and SCFAs whereas data on influence of lactobacilli remain disparate. Therefore, we selected lactobacilli for the present study and included enterococci (otherwise relatively less commonly studied for probiotic activity) owing to the intended higher levels of SCFAs, especially butyrate, following its combination with lactobacilli.

## Conclusions

We herein develop a novel human-origin probiotic cocktail containing 5 *Lactobacillus* and 5 *Enterococcus* strains with a potential to inhibit the growth of uropathogenic strains of *Enterobacteriaceae*. Acute and chronic feeding of these selected probiotics also modulates the fecal microbiome and enhances the production of SCFAs in mouse gut and human feces. This work provides evidence that such human-origin probiotics could be exploited as biotherapeutic regimens for human diseases associated with gut microbiome dysbiosis and decreased SCFAs production in the gut. Our data should be useful for future studies aimed at investigating the influence of probiotics on human microbiome, metabolism and associated diseases.

## Methods

### Culture, selection, isolation and characterization of lactobacilli and enterococci

Baby diapers (unidentified) containing fecal samples were collected from bins of the Bright Horizon day care center (Winston-Salem, NC). Fecal samples from 34 individual diapers (0.5 g) were resuspended in 5 mL of MRS medium (to isolate *Lactobacillus* strains) and LM17 supplement with 35 mM lactic acid in M17 medium (for *Enterococcus* strain selection), and incubated at 37 °C for 24 h. Subsequently, cell cultures were serially diluted, spread onto the corresponding selection medium agar plates, and at least 10 colonies from each sample were picked up after incubation at 37 °C for 12–24 h. Colonies were further purified by the streak plate method. We performed Gram staining^52^ to exclude gram-negative bacteria. We detected catalase activity using 30% of hydrogen peroxide and recording bubble formation, to eliminate gas-forming isolates.

### PCR for virulence genes

Gram-positive and catalase-negative colonies were screened for the presence of virulence genes by PCR, using primers for various common virulence genes listed in Supplementary Table S5. Virulent genes including *agg*, *gelE*, *cylM*, *cylB*, *cylA*, *esp*, *efaAfs*, *efaAfm*, *cpd*, *cob*, *ccf* and *cad* were detected for *Enterococcus* and *Streptococcus* strains as described by Eaton and Gasson^53^. For *Lactobacillus* strains, besides *gelE*, *esp*, *cylA* and *efaAfs*, other virulent genes *hyl*, *asa*, *ace*, *hdc*, *tdc* and *odc* were included and detected as reported by Casarotti *et al*.^54^. PCR was carried out in a 20-µL reaction mixture with MyTaq™ Red Mix (Bioline) buffer. PCR products were detected on 1.0% agarose gel electrophoresis and using gel imaging system (Kodak Image Station 4000 R; Carestream Health Inc., Rochester, NY).

### Antibiotic susceptibility assay

Antibiotic susceptibility was determined by disc diffusion tests using antimicrobial susceptibility test discs (BBL™ Sensi-Disc™, BD Life Sciences, USA) following the manufacturer’s instructions. *Lactobacillus* and *Enterococcus* strains were cultivated overnight in MRS and LM17, respectively, and 50 µL of cultures were spread onto MRS or LM17 agar plates. Antibiotic-impregnated discs containing tetracycline (30 µg), chloramphenicol (30 µg), kanamycin (30 µg), erythromycin (15 µg), rifampicin (30 µg), vancomycin (30 µg), gentamycin (10 µg), streptomycin (10 µg), amoxicillin (25 µg), or ampicillin (10 µg) were applied to agar plates inoculated with bacterial strains. After being cultivated at 37 °C for 16–18 h, the diameterd of complete inhibition zones were measured. Antibiotic susceptibility was recorded as resistant (R), intermediate (MS) and susceptible (S) according to the definition based on the zone diameter in the manufacturer’s manual.

### Genetic identification of isolated strains

The selected isolates were confirmed for their genus- and species-level identity by sequencing of the 16S rRNA gene. The 16S rDNA from the selected isolates was amplified by colony PCR with the 27 F and 1492 R universal primers (27 F: 5′-AGAGTTTGATCCTGGCTCAG-3′ and 1492 R: 5′-GGTTACCTTGTTACGACTT-3′). The amplification mixture (20 μL) contained 10 μL PCR buffer (MyTaq^®^ Red Mix 2×, Bioline, USA), 1 μL (10 μM) of each primer and 7μL of nuclease-free water. Single pure colony was picked up and added to 10 μL sterile ultra-pure distilled water (InVitrogen) and 1 μL of this suspension was added as a template into the PCR reaction mix. The PCR reaction conditions were: 95 °C for 4 min; 30 cycles of 95 °C for 30 s, 55 °C for 30 s and 72 °C for 2 min; and extension at 72 °C for 10 min. Controls devoid of any template DNA were included in the amplification process to serve as a negative control. The integrity of the PCR products was verified by detection of single bands following electrophoresis on 1.0% agarose. The sequencing of amplified 16S rRNA gene from isolates was performed at GeneWiz LLC (NJ, USA); the resulting sequence data were aligned and analyzed using BLAST (http://blast.ncbi.nlm.nih.gov/Blast.cgi) and the identification was confirmed on the basis of the highest hit scores (Supplementary Table S1).

### Probiotic attributes

#### Tolerance to low pH and bile salts

The resistance of short-listed bacterial isolates to low pH and different bile salt concentrations (Oxgall, Sigma-Aldrich, USA) was examined by monitoring the bacterial growth, as per our published methods^55^. Briefly, 900 μL of MRS or LM17 broth, which was adjusted to pH 3.0 or 7.0 (control) or supplemented with 0.3% (w/v) bile (Oxgall; Sigma-Aldrich), was inoculated with 100 μL of an overnight grown culture (≈10^9^ cfu/ml), previously washed twice with PBS (pH 7.2). After incubation for 3 h (pH tolerance) or 4 h (bile tolerance) at 37 °C, 50 μL of serially diluted cultures was spread onto MRS or LM17 agar plates and incubated for 24 h at 37 °C, followed by colony counting (CFU_1_). An aliquot of bacterial suspension was also collected and plated on MRS or LM17 agar plates (CFU_0_) prior to inoculation into the broth. The percentage of viable bacteria was calculated as: Survival (%) = CFU_1_/CFU_0_ × 100.

#### Cell surface hydrophobicity

The hydrophobicity of isolates to hydrocarbons was examined as per the method described earlier^55^. Briefly, the overnight grown culture was centrifuged at 5000 g for 10 min. The cell pellet was washed twice with PBS (pH 7.2), resuspended in PBS, and the initial absorbance was adjusted to 0.7 at 600 nm (Abs_0_). Three mL of bacterial cell suspension was mixed with 0.6 mL of n‐hexadecane (5:1), vortexed for 2 min, and incubated at room temperature for 1 h. Following incubation and phase separation, the aqueous phase was decanted carefully and was subjected to the absorbance measurement (Abs_1_) at 600 nm. The surface hydrophobicity was calculated as per the following formula: Hydrophobicity (%) = (1 − A_1_/A_0_) × 100. Only those strains with considerable hydrophobicity and acid and bile tolerance were subjected to subsequent assays, including tolerance to simulated gastric and intestinal fluids and adherence to Caco-2 cells.

#### Tolerance to simulated gastric and intestinal fluids

Bile tolerance of selected isolates was examined as per the method described elsewhere^56^. Briefly, simulated gastric fluid was prepared by suspending (3 g/L) pepsin in 10 mL of sterile saline solution (0.85% NaCl, w/v). The pH was adjusted to 2.5. Cultures grown overnight (≈10^8^ cfu/mL) were inoculated into the simulated gastric fluid and incubated for 3 h at 37 °C. Simulated intestinal fluid was also prepared by dissolving bile salts (0.3% w/v) and pancreatin (1 mg/mL) in 10 mL of sterile saline solution (0.85% NaCl, w/v). The pH was adjusted to 8.0. Cultures grown overnight (≈10^8^ cfu/mL) were inoculated into the simulated intestinal fluid and incubated for 6 h at 37 °C. Viable counts were determined by serial-dilution and plating of the cultures on MRS or LM17 agar and incubation for 24 h at 37 °C.

#### Adhesion to Caco-2 cells

The assay for bacterial adhesion to Caco-2 cells was performed as per the previously described method^57,58^. Briefly, the cells were grown in 12-well tissue-culture plates in Dulbecco’s modified Eagle’s medium (DMEM; Sigma-Aldrich) supplemented with 10% (v/v) heat-inactivated fetal calf serum (Invitrogen) and penicillin–streptomycin (IU/mL or μg/mL; Sigma-Aldrich). The cells were cultured at 37 °C in an atmosphere of 5% (v/v) CO_2_ and 95% air until a confluent (≈80%) monolayer was reached. Before the adhesion assay, the Caco-2 cell monolayers were washed twice with sterile phosphate-buffered saline (D-PBS, pH 7.4) and were suspended in antibiotic- and serum-free DMEM (2 mL per well) and incubated in 5% CO_2_ at 37 °C for 30 min. The overnight grown probiotics bacteria were washed with PBS and suspended in 1 ml of antibiotic- and serum-free DMEM (≈10^8^ cfu/mL). An aliquot of 100μL of this bacterial suspension was added to each well of the 12-well plate containing Caco-2 cells. The plates were incubated for 2 h at 37 °C in a 5% (v/v) CO_2_ atmosphere. After the incubation, each well was washed five times with PBS (pH 7.4) and 1 mL of trypsin-EDTA (0.25%, Sigma-Aldrich, USA) was added to each well. After incubating the plate for 15 min at room temperature, the detached cells were gently aspirated several times to obtain a homogenous suspension. Finally, the cell suspension was serially diluted with PBS and a 50μL-aliquot of diluted homogenate was spread onto MRS or LM17 agar and incubated at 37 °C for 24 h. Bacterial colonies were counted (X_1_ cfu/mL). Bacterial cells originally added to each well of 12-well plates were also counted prior to seeding into the plate (X_0_ cfu/mL). The adhesion was calculated as: adhesion (%) = (X_1_/X_0_) × 100. The assays were performed in three independent experiments.

#### Anti-pathogenic activity

LB (lysogeny broth) medium was used to grow pathogenic strains *E. coli* CFT073 and *K. pneumoniae KPPR*1. However, these strains were also tested for growth on MRS and LM17 and inhibitory effect of probiotic strains was tested through well diffusion and spot-on-lawn assays as described by Aspri *et al*.^59^ with minor modifications. Cultures of five *Lactobacillus* and five *Enterococcus* strains after 24 h cultivation were centrifuged at 12,000 g for 10 min. Supernatants were passed through 0.22 µm cellulose acetate membrane filters (2×) to obtain cell free samples and 150 μL of each supernatant was added into the holes dug on the LB agar plate spread with 50 μL of the pathogenic strain. The plates were incubated at 37 °C overnight, and inhibition zone diameters were recorded. In the spot-on-lawn assay, 10 μL of cell culture from each strain was used to spot on MRS or LM17 agar plates spread with indicator strains, and plates were cultivated overnight to check inhibition zones. Hydrogen peroxide strip analyses were performed using peroxide test strips (Quantofix® Peroxides-100; Sigma-Aldrich) according to the manufacturer’s instructions. Furthermore, heat sensitivity of the active substance was tested by comparing the antimicrobial activity before and after being treated at 70 °C for 1 h. Cell free supernatant was incubated with proteinase K (0.1 mg/mL) and testing the residual activity after 2 h through a well diffusion assay against *E. coli* CFT073 to determine involvement of proteinacious components in antimicrobial activity.

### Mice studies

#### Preparation of probiotics for animal treatment

Pure probiotic strains were grown in corresponding media for 6–8 h to achieve the peak of log phase, and washed twice with PBS to remove traces of culture media. Equal counts (1 × 10^11^ CFU) of freshly prepared bacterial cells were pooled in three groups; (1) *Lactobacilli*: containing five selected strains of lactobacilli only; (2) *Enterococci*: containing five selected strains of enterococci only; and (3) *Probiotic cocktail*: containing all 10 strains i.e., five lactobacilli and five enterococci. The final dose (1 × 10^11^ CFU/mice/dose) was chosen on the basis of published literature^60–62^ and was optimized in 300 μL of PBS for oral gavage.

#### Single-dose study

C57BL/6 J mice were randomly divided into four groups (n = 6 per group): (1) *control*, (2) *lactobacilli*, (3) *enterococci* and (4) *probiotic cocktail*. Group 2, 3 and 4 were given a single dose of 1 × 10^11^ CFU/mice of lactobacilli, enterococci, and probiotic cocktail mixtures, respectively, by gavage. Control group was given equal volume of sterile PBS. Fecal samples were collected before treatment (0 h) and after 8 h, 1 day (24 h), 3 days and 10 days to analyze gut microbiome and SCFAs content.

#### Five-dose study

Similar to single dose study, C57BL/6J mice were randomized in four groups as described above and treated once-daily with 1 × 10^11^ CFU/mice of lactobacilli, enterococci and probiotics cocktail mixtures for 5 consecutive days by gavage; the control group was given equal volumes of sterile PBS once-daily by gavage for 5 days. Fecal samples were collected before treatment (0 h), after 5 days, 2, 3 and 5 weeks (i.e., 1, 2 and 4 weeks post-treatment).

All mice in both studies were allowed to eat normal chow and drinking water *ad-libitum* throughout the study. Body weight, food and water intakes were measured before treatments and at each time-point when fecal samples were collected in both studies. However, no significant differences were observed in any measures throughout the study period (Supplementary Fig. S9a,b). All the animal studies were conducted following procedures approved by the Wake Forest School of Medicine, Animal Research Program’s Institutional Animal Care and Use Committee (IACUC).

### Fecal slurry fermentation assay

The culture media used for *in-vitro* fecal fermentation was prepared as described by Boler *et al*.^63^. Fecal samples were collected from 2 healthy donors, and snap-frozen in liquid nitrogen followed by storage at −80 °C until further use. Fecal collection is approved by Wake Forest School of Medicine’s Institutional Review Board. In anaerobic chamber, fecal samples were thawed, diluted (1:10 w/v) in anaerobic dilution solution (Nacl 5; glucose 2; Cystein-Hcl 0.3; g/L) and vortexed for 15 minutes for complete homogenization. The mixture was filtered through four layers of cheesecloth and was immediately used for inoculation of tubes containing media inoculated with probiotics. The final fecal suspension was divided into four groups: (a) control: fermentation media without probiotics, (b) lactobacilli-alone, (c) enterococci-alone, and (d) probiotic cocktail. Each probiotic combination (1 × 10^6^ CFU/mL) was added to 26 mL fermentation media in sterile 50-mL falcon tubes. The tubes were kept inside the anaerobic chamber for 24 h to allow hydration of samples before starting the fermentation. Four mL of the freshly prepared fecal inoculum was added to each tube, and transferred to the built-in incubator in an anaerobic chamber at 37 °C with periodic mixing for 24 h. Samples were taken at 0, 9 and 24 h during fermentation, and pH of samples was measured using laboratory pH meter. The samples were centrifuged at 14000 g for 10 minutes at 4 °C; the supernatant was immediately frozen for SCFAs analysis while the pellets were stored at −80 °C for microbiome analyses. All experiments were repeated twice, each time in triplicate.

### Fecal microbiome analysis

We conducted 16S rRNA gene amplification and sequencing as per our previously described methods^64^. In brief, nearly 200 mg of mice feces or fecal slurry pellets were used to extract genomic DNA using the Qiagen DNA Stool Mini Kit (Qiagen, CA, USA) per the manufacturer’s instructions. We modified the lysis temperature to 95 °C instead of 75 °C (as recommended by the manufacturer) for more efficient lysis and DNA yield of gram-positive bacteria. The V4 region of bacterial 16S rRNA gene was amplified using the primers 515 F (barcoded) and 806 R in accordance with the Earth Microbiome Project protocol^65^, with a minor modification as described in our previous reports^64^. The resulting amplicons were purified with AMPure^®^ magnetic purification beads (Agencourt) and the purified products quantified using the Qubit-3 fluorimeter (InVitrogen). Equal amounts of purified PCR products were pooled; the resulting pool was quantified, normalized to 4 nm, denatured and diluted to 8 pM, and sequenced on an Illumina MiSeq sequencer (using Miseq reagent kit v3). The sequences were de-multiplexed, quality filtered, clustered, and analyzed using the Quantitative Insights into Microbial Ecology (QIIME, version 1.9.1) software. To avoid bias due to different sequencing depth, the OTU tables were rarefied to the lowest number of sequences per sample (single-dose study: 6650 sequences per sample; 5-dose study: 3800 sequences per sample; human fecal slurry study: 20,000 sequences per sample) for computing alpha-diversity metrics within QIIME. Linear discriminant analysis (LDA) analysis and cladograms were developed on genus level data using LDA effect size (LefSe)^66^ on Galaxy platform (https://huttenhower.sph.harvard.edu/galaxy/). Each dot in the PCA analysis of the gut microbiome signature represents an independently pooled equal amount of DNA of 2 mice from same group and three independent experiments of human fecal slurry.

### SCFA analysis

To determine the organic acid/SCFA production of the screened isolates, overnight cultures of *Lactobacillus* and *Enterococcus* strains were inoculated into MRS/LM17 medium (inoculum of 1% and 2%, respectively). Through 24 h cultivation at 37 °C, supernatants were collected after centrifugation (12,000 g for 10 min), followed by passing through membrane filter. SCFAs from fecal slurry supernatant were obtained through centrifugation (12,000 g for 10 min) twice, and the supernatant was passed through a 0.45 µm membrane filter. SCFAs were extracted from fecal samples by taking 50 mg feces in a 1.5 mL Eppendorf tube, grinding the slurry with a pellet pestle motor, and suspending it in 1 mL PBS buffer (0.1 M, pH 7.4), followed by vortexing for 1 min at 20-min intervals for about 4 h. Thereafter, samples were immediately centrifuged and passed through a 0.45 µm membrane filter. Cell-free samples were used for determining concentrations of SCFAs (lactate, acetate, propionate and butyrate) using a high-performance liquid chromatography (Waters-2695 Alliance HPLC system, Waters Corporation, Milford, MA, USA) with DAD detector at 210 nm, equipped with an Aminex HPX-87H column (Bio-Rad Laboratories, Hercules, CA). Samples (10 μL) were injected each time and H_2_SO_4_ (0.005 N) was used to elute the column with a flow rate of 0.6 mL/min at 35 °C.

### Statistical analyses

OTUs with abundances higher than 1% were included in the subsequent analyses. Taxonomy assignment and diversity analyses were computed within QIIME to compare richness of bacterial species among experimental groups. Alpha-diversity (rarefaction curve for observed OTUs, Chao1, PD Whole Tree and Shannon) indices were computed with core_diversity_analysis.py script. Beta-diversity was generated within QIIME by using weighted and unweighted Unifrac distance matrices. Principal components analysis was performed (using EMPeror version 0.9.3-dev) to determine the influence of treatments on the overall microbiome composition. Bacterial diversity and abundance between groups within the same study were compared using non-parametric tests in R statistical software package (version 3.4.3; https://www.r-project.org/). Statistically significant differences between different groups were calculated by Kruskal-Wallis test followed by Dunn’s post-hoc analysis. SCFA contents were compared among groups by unpaired t-test. Unless otherwise stated, all assays were performed in either triplicate or three independently repeated experiments, and all the values presented here are means ± SEM. P < 0.05 was considered statistically significant.

### Availability of data and materials

The datasets used and/or analyzed during the current study are available from the corresponding author on reasonable request.

### Ethics approval and consent to participate

All protocols and procedures related to the sampling, care, and management of animals were reviewed and approved by the Institutional Animal Care and Use Committee at the Wake Forest School of Medicine. All experiments and samplings were carried out in accordance with ethical and biosafety protocols approved by Institutional guidelines.

## Electronic supplementary material

Supplementary Information

Supplementary Table S6

Supplementary Table S7

Supplementary Table S8
