# Supplementary material for: Human-origin probiotic cocktail increases short-chain fatty acid production via modulation of mice and human gut microbiome
Source: Sci Rep. 2018 Aug 23;8:12649. doi: 10.1038/s41598-018-30114-4 (PMC6107516; doi:10.1038/s41598-018-30114-4)
Supplement: Supplementary file 1 — Supplementary Information [file 41598_2018_30114_MOESM1_ESM.pdf]

Supplementary Information

Human-origin probiotic cocktail increases short-chain fatty acid production via modulation of mice and human gut microbiome

Ravinder Nagpal, Shaohua Wang, Shokouh Ahmadi, Joshua Hayes, Jason Gagliano, Sargurunathan Subashchandrabose, Dalane W Kitzman, Thomas Becton, Russel Read, and Hariom Yadav

LEGENDS OF SUPPLEMENTARY FIGURES

Supplementary figure S1. Representative images of agar plates analyzed for anti-pathogenic potential of selected probiotics against uropathogenic *E. coli* and *Klebsiella pneumoniae*. a) Representative plates showing well-diffusion assay using selected probiotics against *E. coli* (upper lane) and *K. pneumoniae*. b) Antimicrobial activity analyzed by agar-spot test. c) Quantification of zone of inhibition of selected probiotics against *E. coli* using agar spot test. All these assays were repeated 3-5 times independently and were analyzed by a person blinded to group assignments. Graph bars represent mean of values of 3-5 replicates/ independent experiments and error bars represent standard error of means.

Supplementary figure S2. Hydrogen peroxide (H_2O_2) detection in probiotic culture supernatants. Representative images of only one peroxide test strip (Quantofix® Peroxides-100; Sigma-Aldrich) from each sample of probiotic supernatants and controls are shown. Assays was performed in 3-4 replicates in independent tubes and/or experiments.

Supplementary figure S3. Changes in mouse gut microbiome after a single dose of probiotic treatment. Changes in mouse gut microbiome alpha diversity indices including PD whole tree (a), Chao1 (b), and observed OTUs (c), and in relative abundance of major phyla including Bacteroidetes (d), Firmicutes (e) and Verrucomicrobia (f) and major genera (g) after single-dose treatment with selected probiotic lactobacilli and enterococci as well as their combination (cocktail); followed up for up to 10 days post-treatment.

Supplementary figure S4. **Linear discriminant analysis results after 1 day of probiotic treatment.** Effect size (LEfSe) analysis showing LDA scores reflecting changes in the abundance of major microbial taxa following a single dose of lactobacilli (a), enterococci (b), or the combined probiotic mixture; c) compared to control group at 1-day post-treatment.

Supplementary figure S5. Impact of 5 dose probiotics treatments on the mouse gut microbiome. Changes in mouse gut microbiome alpha diversity indices including PD whole tree (a), Chao1 (b), and observed OTUs (c), and in relative abundance of major genera (d), as well as relative abundance of major phyla like Bacteroidetes (e), Firmicutes (f), Proteobacteria (g) and Verrucomicrobia (h) after five-dose treatment with selected probiotic lactobacilli and enterococci as well as their combination (cocktail); followed up for up to 5 weeks post-treatment.

Supplementary figure S6. Effects of five-dose probiotics treatments on linear discriminant analysis (LDA) of gut microbiome. Effect size (LEfSe) analysis showing LDA scores reflecting changes in the abundance of major microbial taxa following a five-dose of lactobacilli (a), enterococci (b), or the combined probiotic mixture; c) compared to control group after 5 days of post-treatment.

Supplementary figure S7. Human gut microbiome changes in fecal slurry culture system. Changes in gut microbiome alpha-diversity indices including PD whole tree (a), Chao1 (b), and observed OTUs (c) and major genera (d) during 24 h of probiotic treatment.

Supplementary figure S8. Linear discriminant analysis results after 9 hours of inoculation. Effect size (LEfSe) analysis showing the LDA score of changes in the abundance of major microbial taxa after 9 h of inoculation with probiotic lactobacilli (a), enterococci (b) and the combined probiotic mixture; c) compared to non-treated control.

Supplementary figure S9. Body weight (a) and food intake (b) after during and after five-dose treatments of probiotics in mice. Graph bars represent mean of values n=5-6 animals in each group and error bars represent standard error of means. NS: no-significant differences were observed among groups during student t-test analyses.

Supplementary Figures

Supplementary FIGURE S1

(a) Supernatant-about 150 µl into each well

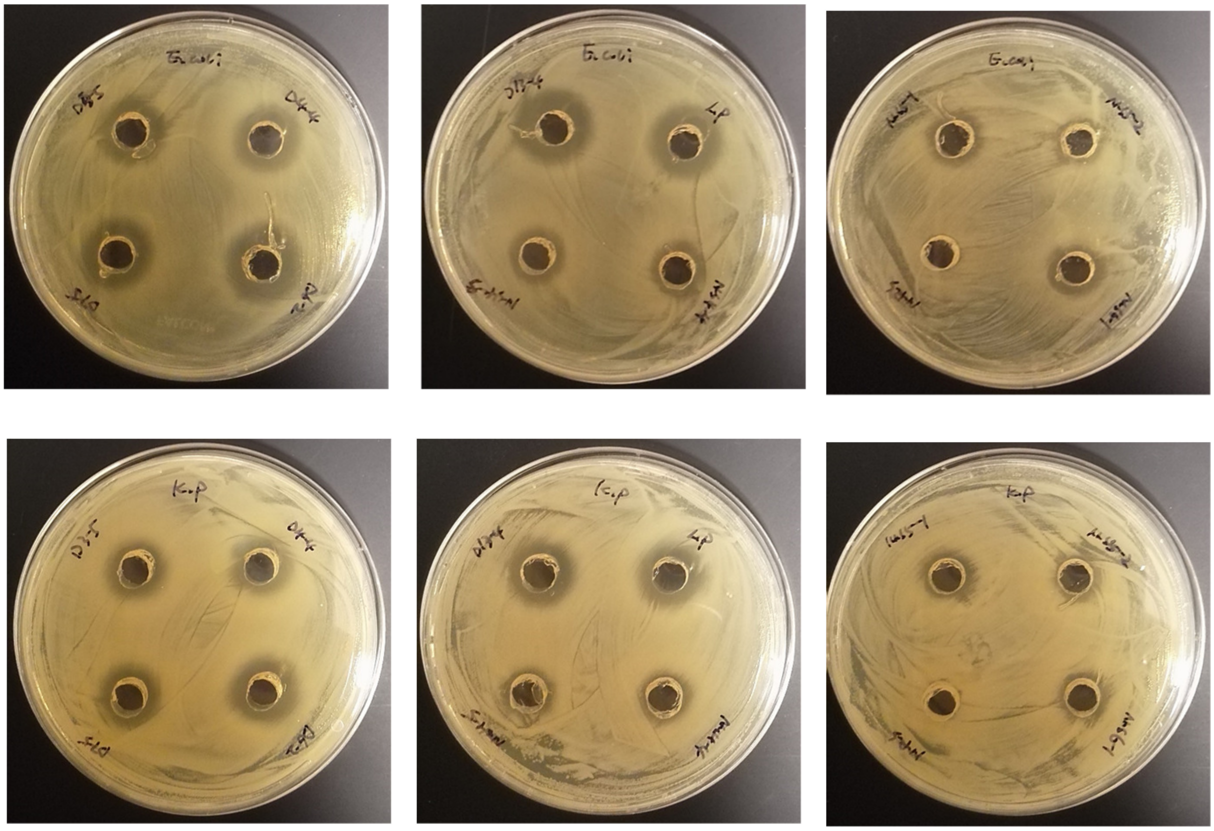

(b) Live cells-drop 5 µl cell culture onto plates spread with *E. coli*

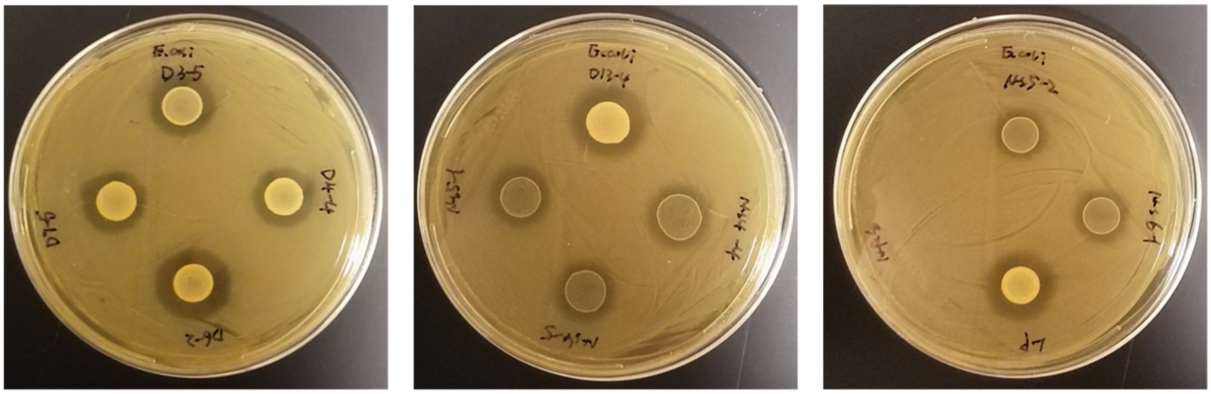

(c)

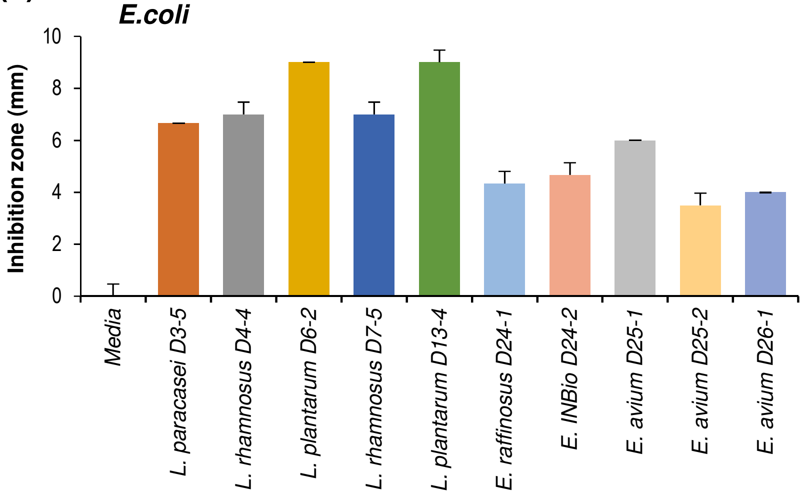

Supplementary FIGURE S2

H_2O_2 detection

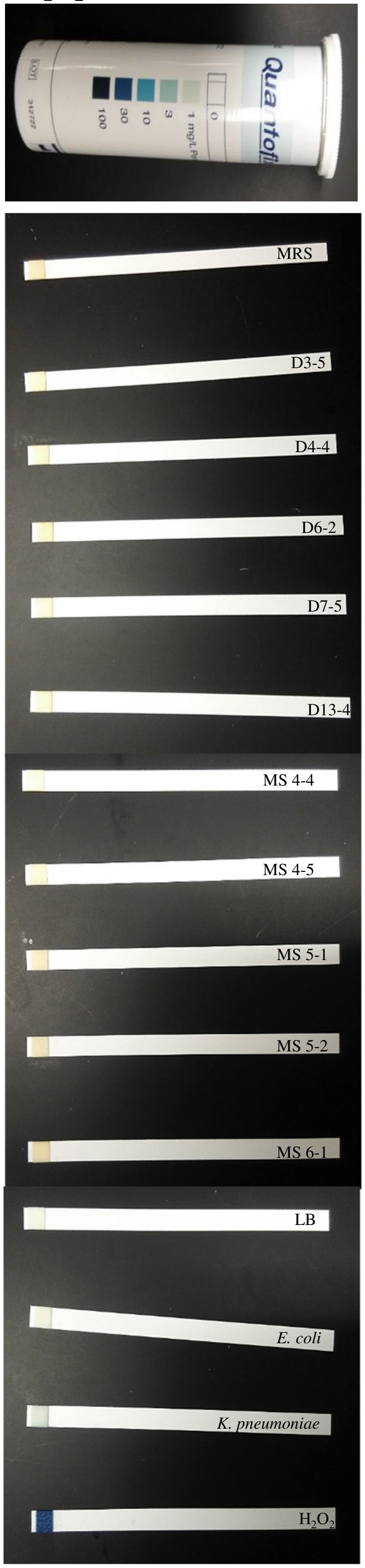

Supplementary FIGURE S3

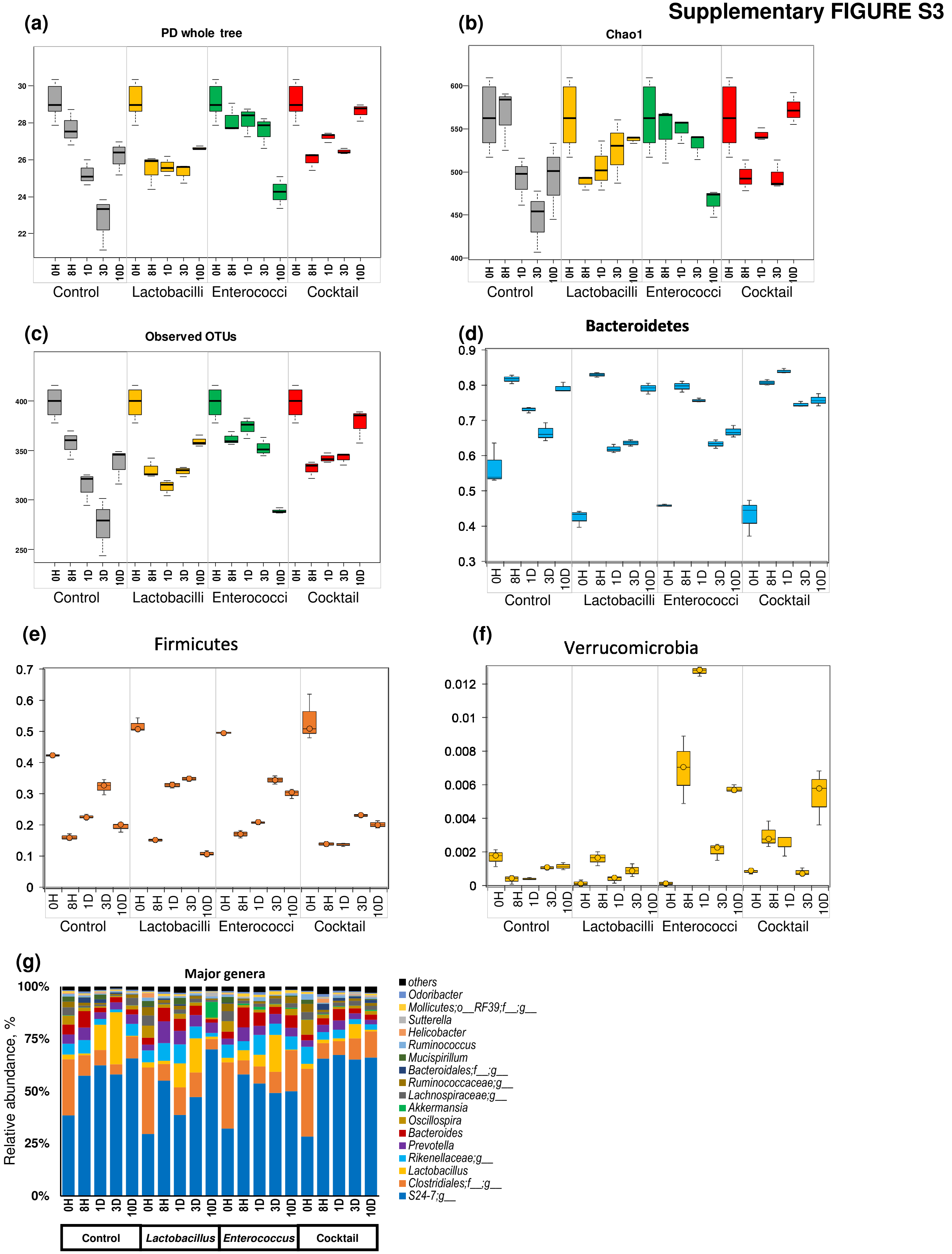

Supplementary FIGURE S4

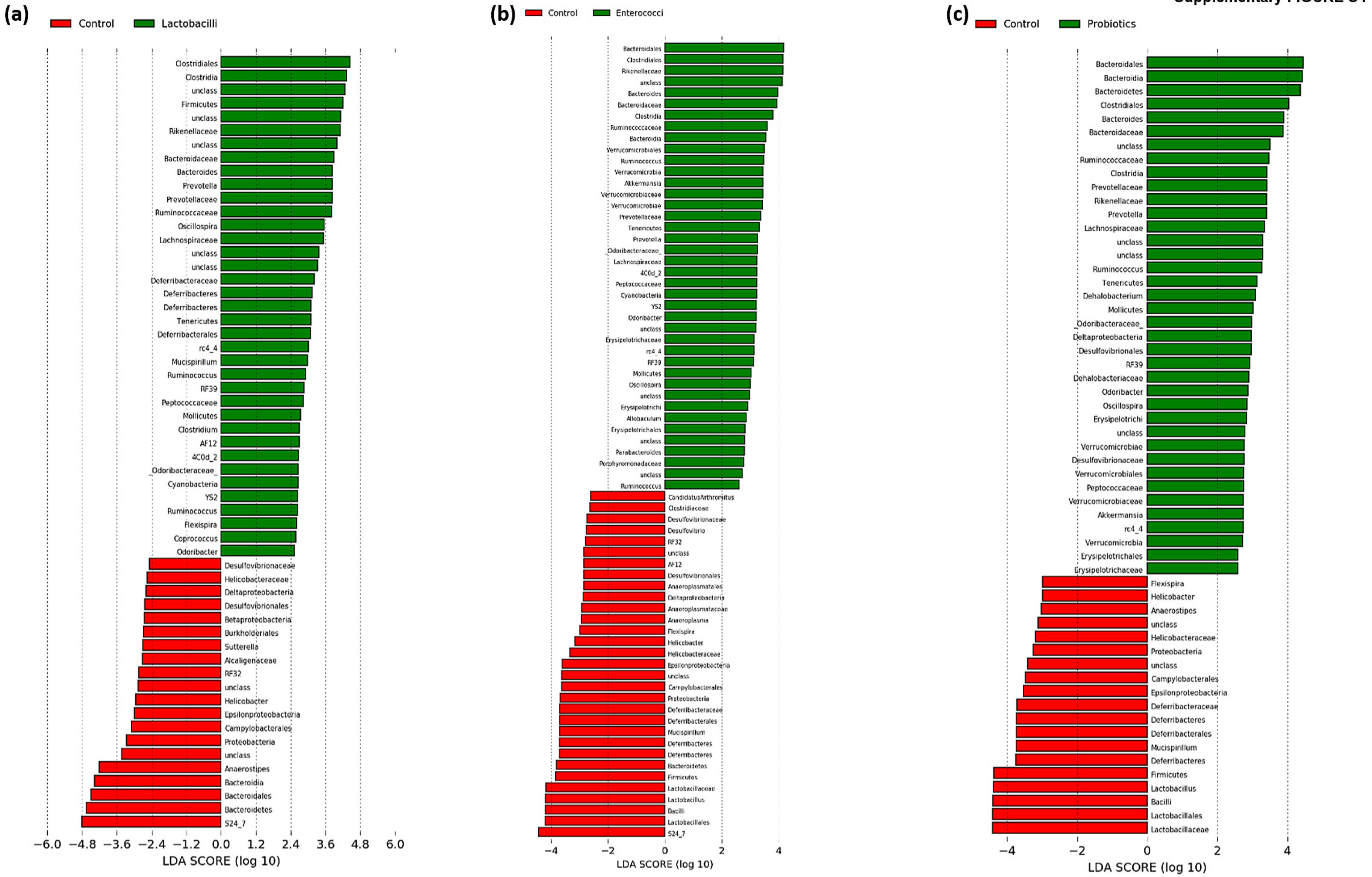

Supplementary FIGURE S5

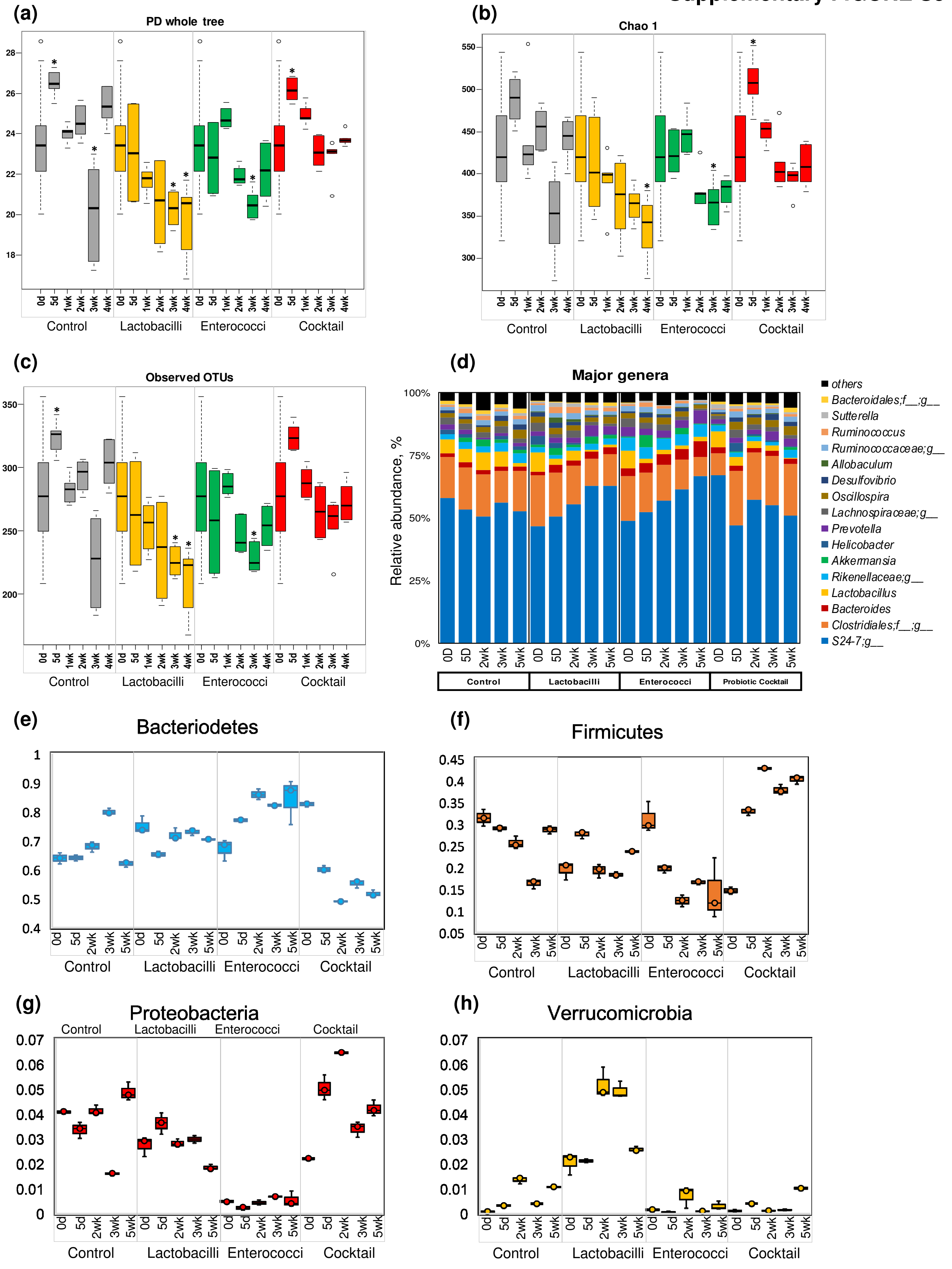

Supplementary FIGURE S6

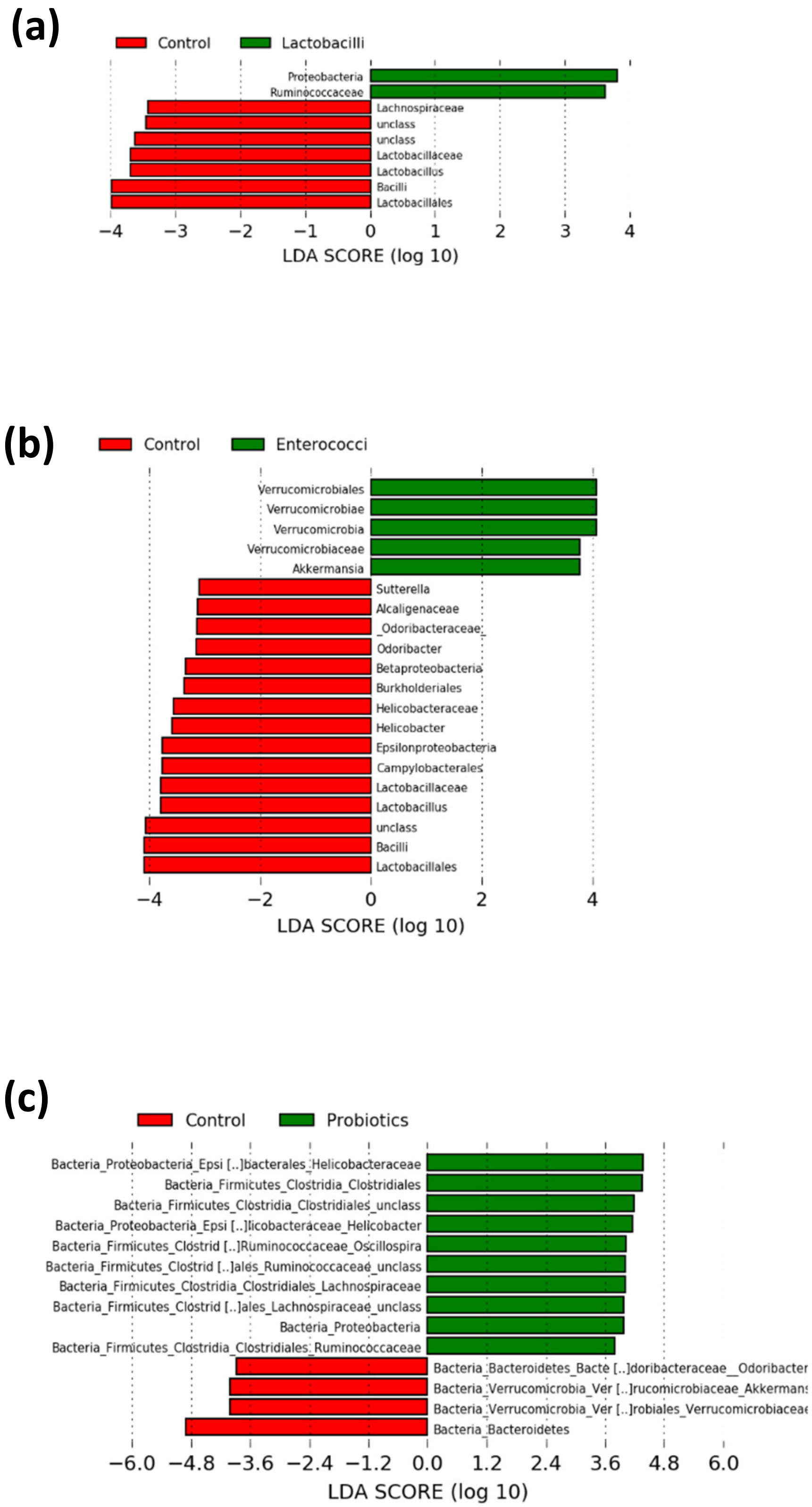

Supplementary FIGURE S7

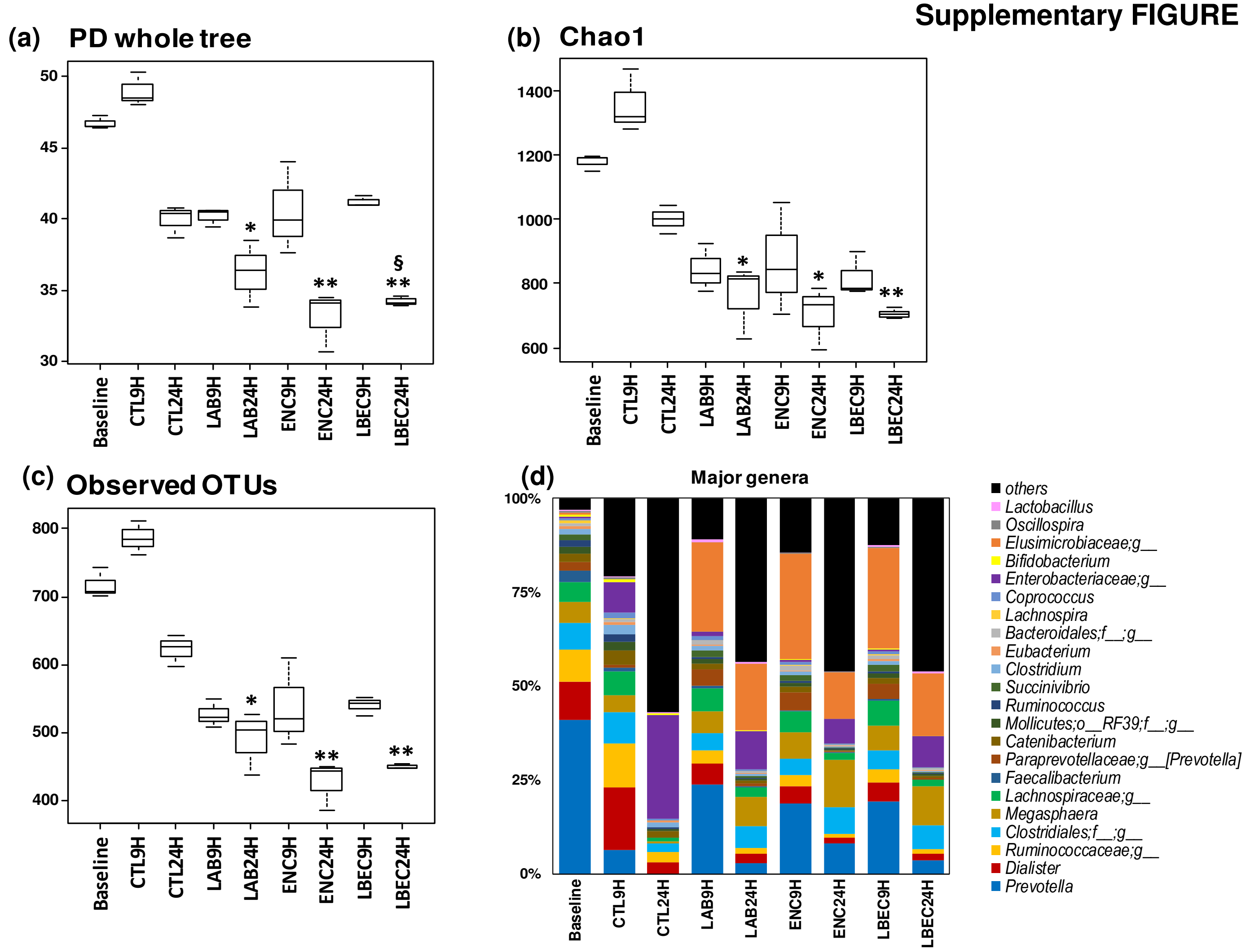

Supplementary FIGURE S8

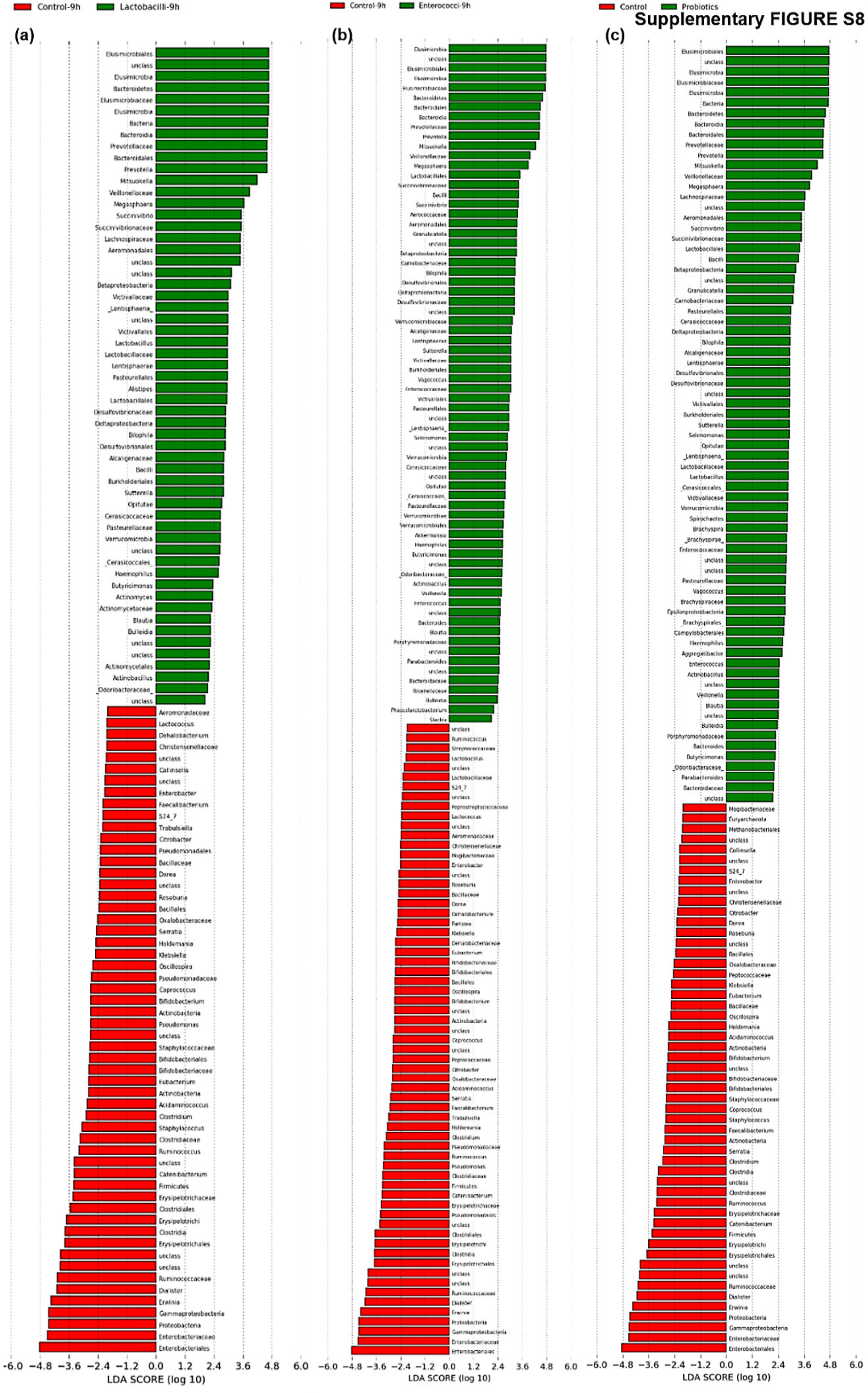

Supplementary FIGURE S9

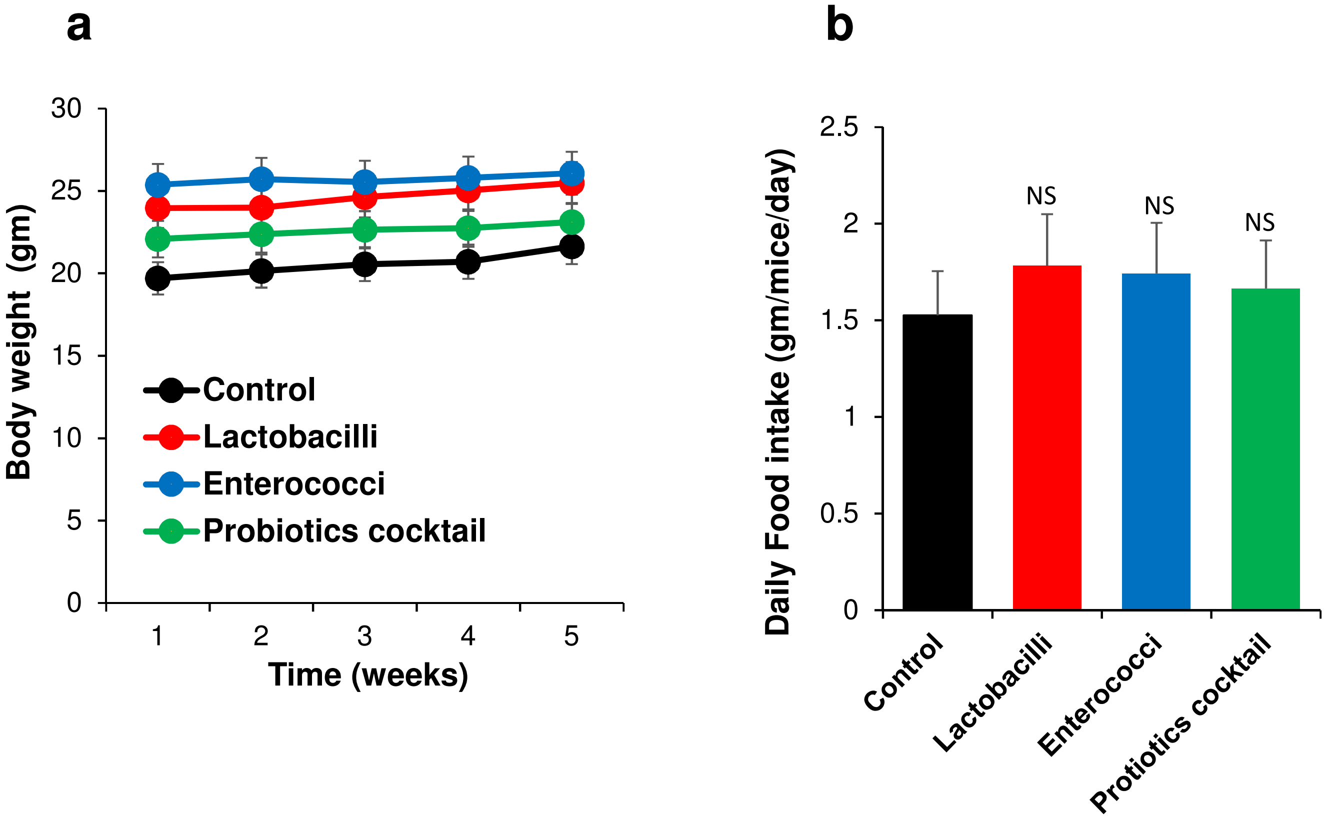

Supplementary table S1: Isolation and identification of isolates cultivated using MRS and LM17 media.

#	Isolates	Gram staining	Catalase activity	Morphology	Identification (16S rDNA sequencing)	Alignment score (%)
**Isolates grown on MRS**						
1	D3-1	+	-	long, slender; rods/bacilli; arranged in short or long chains	*Lactobacillus paracasei*	98
2	D3-2	+	-	long, slender; rods/bacilli; arranged in short or long chains	*Lactobacillus paracasei*	99
3	D3-5	+	-	long, slender; rods/bacilli; arranged in short or long chains	*Lactobacillus paracasei*	99
4	D4-1	+	-	long, slender; rods/bacilli; arranged in short or long chains	*Lactobacillus paracasei*	98
5	D4-2	+	-	long, slender; rods/bacilli; arranged in short or long chains	*Lactobacillus rhamnosus*	99
6	D4-3	+	-	long, slender; rods/bacilli; arranged in short or long chains	*Lactobacillus rhamnosus*	99
7	D4-4	+	-	long, slender; rods/bacilli; arranged in short or long chains	*Lactobacillus rhamnosus*	99
8	D4-7	+	-	long, slender; rods/bacilli; arranged in short or long chains	*Lactobacillus rhamnosus*	96
9	D5-3	+	-	long, slender; rods/bacilli; arranged in short or long chains	*Lactobacillus rhamnosus*	99
10	D5-4	+	-	long, slender; rods/bacilli; arranged in short or long chains	*Lactobacillus rhamnosus*	99
11	D5-6	+	-	long, slender; rods/bacilli; arranged in short or long chains	*Lactobacillus rhamnosus*	99
12	D6-1	+	-	long, slender; rods/bacilli; arranged in short or long chains	*Lactobacillus rhamnosus*	99
13	D6-2	+	-	long, slender; rods/bacilli; arranged in short or long chains	*Lactobacillus plantarum*	99
14	D6-6	+	-	long, slender; rods/bacilli; arranged in short or long chains	*Lactobacillus rhamnosus*	99
15	D7-4	+	-	long, slender; rods/bacilli; arranged in short or long chains	*Lactobacillus rhamnosus*	99
16	D7-5	+	-	long, slender; rods/bacilli; arranged in short or long chains	*Lactobacillus rhamnosus*	98
17	D7-7	+	-	long, slender; rods/bacilli; arranged in short or long chains	*Lactobacillus rhamnosus*	99
18	D8-1	+	-	long, slender; rods/bacilli; arranged in short or long chains	*Lactobacillus rhamnosus*	99
19	D8-4	+	-	long, slender; rods/bacilli; arranged in short or long chains	*Lactobacillus rhamnosus*	99
20	D8-5	+	-	long, slender; rods/bacilli; arranged in short or long chains	*Lactobacillus rhamnosus*	97
21	D9-1	+	-	long, slender; rods/bacilli; arranged in short or long chains	*Lactobacillus rhamnosus*	97
22	D9-2	+	-	long, slender; rods/bacilli; arranged in short or long chains	*Lactobacillus rhamnosus*	98
23	D9-3	+	-	long, slender; rods/bacilli; arranged in short or long chains	*Lactobacillus rhamnosus*	99
24	D9-6	+	-	long, slender; rods/bacilli; arranged in short or long chains	*Lactobacillus plantarum*	99
25	D10-4	+	-	long, slender; rods/bacilli; arranged in short or long chains	*Lactobacillus paracasei*	99
26	D10-6	+	-	long, slender; rods/bacilli; arranged in short or long chains	*Lactobacillus reuteri*	98
27	D10-8	+	-	long, slender; rods/bacilli; arranged in short or long chains	*Lactobacillus rhamnosus*	99
28	D12-3	+	-	long, slender; rods/bacilli; arranged in short or long chains	*Lactobacillus plantarum*	98
29	D12-6	+	-	long, slender; rods/bacilli; arranged in short or long chains	*Lactobacillus rhamnosus*	98
30	D12-7	+	-	long, slender; rods/bacilli; arranged in short or long chains	*Lactobacillus paracasei*	97
31	D12-8	+	-	long, slender; rods/bacilli; arranged in short or long chains	*Lactobacillus paracasei*	96
32	D13-4	+	-	long, slender; rods/bacilli; arranged in short or long chains	*Lactobacillus plantarum*	98
33	D13-7	+	-	long, slender; rods/bacilli; arranged in short or long chains	*Lactobacillus paracasei*	99
34	D17-9	+	-	long, slender; rods/bacilli; arranged in short or long chains	*Lactobacillus paracasei*	98
**Isolates grown on LM17**						
1	D21-1	+	-	Cocci in pairs or chains	*Enterococcus faecium*	99
2	D21-2	+	-	Cocci in pairs or chains	*Enterococcus faecium*	99
3	D21-3	+	-	Cocci in pairs or chains	*Enterococcus* INBio	99
4	D21-4	+	-	Cocci in pairs or chains	*Enterococcus faecium*	99
5	D21-5	+	-	Cocci in pairs or chains	*Enterococcus* INBio	99
6	D24-1	+	-	Cocci in pairs or chains	*Enterococcus raffinosus*	99
7	D24-2	+	-	Cocci in pairs or chains	*Enterococcus* INBio	98
8	D25-1	+	-	Cocci in pairs or chains	*Enterococcus avium*	99
9	D25-2	+	-	Cocci in pairs or chains	*Enterococcus avium*	99
10	D26-1	+	-	Cocci in pairs or chains	*Enterococcus avium*	99
11	D28-1	+	-	Cocci in pairs or chains	*Enterococcus faecalis*	98
12	D28-2	+	-	Cocci in pairs or chains	*Enterococcus avium*	99
13	D21-6	+	-	Cocci in pairs or chains	*Enterococcus gallinarum*	99
14	D22-1	+	-	Cocci in pairs or chains	*Streptococcus infantarius*	99
15	D24-3	+	-	Cocci in pairs or chains	*Enterococcus faecalis*	99
16	D24-4	+	-	Cocci in pairs or chains	*Enterococcus faecalis*	99
17	D25-3	+	-	Cocci in pairs or chains	*Enterococcus raffinosus*	99
18	D25-4	+	-	Cocci in pairs or chains	*Enterococcus raffinosus*	99
19	D26-2	+	-	Cocci in pairs or chains	*Streptococcus lutetiensis*	99
20	D27-1	+	-	Cocci in pairs or chains	*Streptococcus lutetiensis*	99
21	D27-2	+	-	Cocci in pairs or chains	*Streptococcus lutetiensis*	99
22	D21-7	+	-	Cocci in pairs or chains	*Enterococcus faecium*	99
23	D21-8	+	-	Cocci in pairs or chains	*Enterococcus faecium*	99
24	D22-1	+	-	Cocci in pairs or chains	*Enterococcus faecalis*	99
25	D22-2	+	-	Cocci in pairs or chains	*Enterococcus faecalis*	99
26	D24-5	+	-	Cocci in pairs or chains	*Enterococcus faecalis*	99
27	D25-5	+	-	Cocci in pairs or chains	*Streptococcus lutetiensis*	99
28	D25-6	+	-	Cocci in pairs or chains	*Streptococcus lutetiensis*	99
29	D28-3	+	-	Cocci in pairs or chains	*Enterococcus faecalis*	99
30	D24-5	+	-	Cocci in pairs or chains	*Enterococcus faecalis*	99
31	D24-6	+	-	Cocci in pairs or chains	*Enterococcus faecalis*	99
32	D28-4	+	-	Cocci in pairs or chains	*Enterococcus faecalis*	99

Supplementary Table S2. Virulent genes detection in *Lactobacillus* strains.

#	Isolates	Virulent genes									
		gelE	*esp*	*cylA*	*efaAfs*	*hyl*	*asa*	*ace*	*hdc*	*tdc*	*odc*
1	*L. paracasei* D3-1	-	-	-	-	-	-	-	-	-	-
2	*L. paracasei* D3-2	-	-	-	-	-	-	-	-	-	-
3	*L. paracasei* D3-5	-	-	-	-	-	-	-	-	-	-
4	*L. paracasei* D4-1	-	-	-	-	-	-	-	-	-	-
5	*L. rhamnosus* D4-2	-	-	-	-	-	-	-	-	-	-
6	*L. rhamnosus* D4-3	-	-	-	-	-	-	-	-	-	-
7	*L. rhamnosus* D4-4	-	-	-	-	-	-	-	-	-	-
8	*L. rhamnosus* D4-7	-	-	-	-	-	-	-	-	-	-
9	*L. rhamnosus* D5-3	-	-	-	-	-	-	-	-	-	-
10	*L. rhamnosus* D5-4	-	-	-	-	-	-	-	-	-	-
11	*L. rhamnosus* D5-6	-	-	-	-	-	-	-	-	-	-
12	*L. rhamnosus* D6-1	-	-	-	-	-	-	-	-	-	-
13	*L. plantarum* D6-2	-	-	-	-	-	-	-	-	-	-
14	*L. rhamnosus* D6-6	-	-	-	-	-	-	-	-	-	-
15	*L. rhamnosus* D7-4	-	-	-	+	-	-	-	-	-	-
16	*L. rhamnosus* D7-5	-	-	-	-	-	-	-	-	-	-
17	*L. rhamnosus* D7-7	-	-	-	-	-	-	-	-	-	-
18	*L. rhamnosus* D8-1	-	-	-	-	-	-	-	-	-	-
19	*L. rhamnosus* D8-4	-	-	-	-	-	-	-	-	-	-
20	*L. rhamnosus* D8-5	-	-	-	-	-	-	-	-	-	-
21	*L. rhamnosus* D9-1	-	-	-	-	-	-	-	-	-	-
22	*L. rhamnosus* D9-2	-	-	-	-	-	-	-	-	-	-
23	*L. rhamnosus* D9-3	-	-	-	-	-	-	-	-	-	-
24	*L. plantarum* D9-6	-	-	-	-	-	-	-	-	-	-
25	*L. paracasei* D10-4	+	+	+	+	-	+	+	-	-	-
26	*L. reuteri* D10-6	-	-	-	-	-	-	-	-	-	-
27	*L. rhamnosus* D10-8	-	-	-	-	-	-	-	-	-	-
28	*L. plantarum* D12-3	-	-	-	-	-	-	-	-	-	-
29	*L. rhamnosus* D12-6	-	-	-	-	-	-	-	-	-	-
30	*L. paracasei* D12-7	-	-	-	+	-	-	-	-	-	-
31	*L. paracasei* D12-8	+	+	+	-	-	+	+	-	-	-
32	*L. plantarum* D13-4	-	-	-	-	-	-	-	-	-	-
33	*L. paracasei* D13-7	-	+	-	+	-	-	-	-	-	-
34	*L. paracasei* D17-9	-	+	-	+	-	-	-	-	-	-

Supplementary Table S3. Virulent genes detection in *Enterococcus* and *Streptococcus* strains.

#	Isolates	Virulent genes											
		agg	***gelE***	***cylM***	***cylB***	***cylA***	***esp***	***efaAfs***	***efaAfm***	***cpd***	***cob***	***ccf***	***cad***
1	*E. faecium* D21-1	-	-	-	-	-	-	-	+	-	-	-	+
2	*E. faecium* D21-2	-	-	-	-	-	-	-	+	-	-	-	+
3	*E.* INBio D21-3	-	-	-	-	-	+	-	-	-	-	-	-
4	*E. faecium* D21-4	-	-	-	-	-	-	-	+	-	-	-	+
5	*E.* INBio D21-5	-	-	-	-	-	+	-	-	-	-	-	-
6	*E. raffinosus* D24-1	-	-	-	-	-	-	-	-	-	-	-	-
7	*E.* INBio D24-2	-	-	-	-	-	-	-	-	-	-	-	-
8	*E. avium* D25-1	-	-	-	-	-	-	-	-	-	-	-	-
9	*E. avium* D25-2	-	-	-	-	-	-	-	-	-	-	-	-
10	*E. avium* D26-1	-	-	-	-	-	-	-	-	-	-	-	-
11	*E. faecalis* D28-1	+	+	+	+	+	+	+	-	+	+	+	+
12	*E. avium* D28-2	-	+	-	-	-	-	+	-	-	-	-	-
13	*E. gallinarum* D21-6	-	-	-	-	-	-	-	-	-	-	-	-
14	*S. infantarius* D22-1	-	-	-	-	-	+	-	-	-	+	+	-
15	*E. faecalis* D24-3	+	+	+	+	-	+	+	-	+	+	-	+
16	*E. faecalis* D24-4	+	+	+	+	-	+	+	-	+	+	-	+
17	*E. raffinosus* D25-3	-	-	-	-	-	-	-	-	-	-	-	-
18	*E. raffinosus* D25-4	+	+	-	+	-	+	+	-	+	+	-	+
19	*S. lutetiensis* D26-2	-	-	-	-	-	+	-	-	+	-	+	+
20	*S. lutetiensis* D27-1	-	-	-	-	-	+	-	-	-	-	-	+
21	*S. lutetiensis* D27-2	-	+	-	-	-	+	+	-	+	-	+	+
22	*E. faecium* D21-7	-	-	-	-	-	-	-	+	-	-	-	+
23	*E. faecium* D21-8	-	+	-	-	-	-	+	+	+	+	+	+
24	*E. faecalis* D22-1	-	-	-	-	-	+	+	+	+	-	+	+
25	*E. faecalis* D22-2	+	+	-	-	-	-	+	-	+	+	+	+
26	*E. faecalis* D24-5	+	+	+	+	+	+	+	-	+	+	+	+
27	*S. lutetiensis* D25-5	-	-	-	-	-	+	-	-	-	-	+	-
28	*E. faecalis* D25-6	-	-	-	-	-	-	-	-	+	-	-	-
29	*E. faecalis* D28-3	+	+	+	+	+	+	+	-	+	-	-	+
30	*E. faecalis* D24-5	+	+	+	+	+	+	+	-	+	+	-	+
31	*E. faecalis* D24-6	+	+	+	+	+	+	+	-	+	+	+	+
32	*E. faecalis* D28-4	+	+	+	+	+	+	+	-	+	+	+	+

Supplementary Table S4. Antibiotic sensitivity analysis of selected strains.

#	Isolates	C30	E15	P10	NB30	TE30	S10	K30	NA30	F/M300
1	*L. paracasei* D3-1	S	S	S	MS	S	R	R	R	R
2	*L. paracasei* D3-2	S	S	S	MS	S	R	R	R	R
3	*L. paracasei* D3-5	S	S	S	S	S	R	R	R	MS
4	*L. paracasei* D4-1	S	S	S	S	S	R	R	R	R
5	*L. rhamnosus* D4-2	S	S	S	S	S	R	R	R	R
6	*L. rhamnosus* D4-3	S	S	S	S	S	R	R	R	R
7	*L. rhamnosus* D4-4	S	S	S	S	S	R	R	R	MS
8	*L. rhamnosus* D4-7	S	S	S	S	S	R	R	R	R
9	*L. rhamnosus* D5-3	S	S	S	MS	S	R	R	R	R
10	*L. rhamnosus* D5-4	S	S	S	S	MS	R	R	R	R
11	*L. rhamnosus* D5-6	S	S	S	MS	S	R	R	R	R
12	*L. rhamnosus* D6-1	S	S	S	S	S	R	R	R	MS
13	*L. plantarum* D6-2	S	S	S	MS	S	R	R	R	MS
14	*L. rhamnosus* D6-6	S	S	S	S	S	R	R	R	MS
15	*L. rhamnosus* D7-5	S	S	S	MS	S	R	R	R	R
16	*L. rhamnosus* D7-7	S	S	S	MS	MS	R	R	R	R
17	*L. rhamnosus* D8-1	S	S	S	S	S	R	R	R	MS
18	*L. rhamnosus* D8-4	S	S	S	S	S	R	R	R	R
19	*L. rhamnosus* D8-5	S	S	S	S	S	R	R	R	R
20	*L. rhamnosus* D9-1	S	S	S	S	S	R	R	R	MS
21	*L. rhamnosus* D9-2	S	S	S	MS	MS	R	R	R	R
22	*L. rhamnosus* D9-3	S	S	S	MS	MS	R	R	R	MS
23	*L. plantarum* D9-6	S	S	S	S	S	R	R	R	MS
24	*L. reuteri* D10-6	S	S	S	S	S	R	R	R	MS
25	*L. rhamnosus* D10-8	S	S	S	S	S	R	R	R	MS
26	*L. plantarum* D12-3	S	S	S	S	S	R	R	R	R
27	*L. rhamnosus* D12-6	S	S	S	MS	MS	R	R	R	MS
28	*L. plantarum* D13-4	S	S	S	MS	S	R	R	R	MS
29	*E. raffinosus* D24-1	S	R	R	MS	R	R	R	R	S
30	*E.* INBio D24-2	S	R	R	MS	R	R	R	R	S
31	*E. avium* D25-1	S	MS	S	MS	R	R	R	R	S
32	*E. avium* D25-2	S	R	S	MS	S	R	R	R	S
33	*E. avium* D26-1	S	R	S	MS	R	R	R	R	S
34	*E. gallinarum D21-6*	S	R	S	S	R	R	R	R	S
35	*E. raffinosus* D25-3	S	R	S	S	R	R	R	R	S

C30, Chloramphenicol 30 µg; NB 30, Novobiocin 30 µg; P10, Penicillin 10 µg; E15, Erythromycin 15 µg; NA30, Nalidixic Acid 30 µg; TE30, Tetracycline 30 µg; S10, Streptomycin 10 µg; K30, Kanamycin 30 µg; F/M 300, Nitrofurantoin 300 µg.

Supplementary Table S5. Primers used for virulent genes detection.

Virulent genes and products	Primers	Sequence (5'-3')
agg (Aggregation protein)	agg-F	AAGAAAAAGAAGTAGACCAAC
	agg-R	AAACGGCAAGACAAGTAAATA
gelE (Gelatinase)	gelE-F	ACCCCGTATCATTGGTTT
	gelE-R	ACGCATTGCTTTTCCATC
cylM (Protein for modification of cytolysin)	cylM-F	CTGATGGAAAGAAGATAGTAT
	cylM-R	TGAGTTGGTCTGATTACATTT
cylB (Protein for transport of cytolysin)	cylB-F	ATTCCTACCTATGTTCTGTTA
	cylB-R	AATAAACTCTTCTTTTCCAAC
cylA (Protein for activation of cytolysin)	cylA-F	TGGATGATAGTGATAGGAAGT
	cylA-R	TCTACAGTAAATCTTTCGTCA
esp (Enterococcal surface protein)	esp-F	TTGCTAATGCTAGTCCACGACC
	esp-R	GCGTCAACACTTGCATTGCCGAA
efaAfs (Cell wall adhesins)	efaAfs-F	GACAGACCCTCACGAATA
	efaAfs-R	AGTTCATCATGCTGTAGTA
efaAfm (Cell wall adhesins)	efaAfm-F	AACAGATCCGCATGAATA
	efaAfm-R	CATTTCATCATCTGATAGTA
cpd (Sex pheromone)	cpd-F	TGGTGGGTTATTTTTCAATTC
	cpd-R	TACGGCTCTGGCTTACTA
cob (Sex pheromone)	cob-F	AACATTCAGCAAACAAAGC
	cob-R	TTGTCATAAAGAGTGGTCAT
ccf (Sex pheromone)	ccf-F	GGGAATTGAGTAGTGAAGAAG
	ccf-R	AGCCGCTAAAATCGGTAAAAT
cad (Sex pheromone)	cad-F	GGTTACCGTGCAGTCTTTGAA
	cad-R	TTCAACACCTGCAGGTGAGCC
hyl (hyaluronidase)	HYLn1	ACAGAAGAGCTGCAGGAAATG
	HYLn2	GACTGACGTCCAAGTTTCCAA
asa (aggregation substance)	ASA11	GCACGCTATTACGAACTATGA
	ASA12	TAAGAAAGAACATCACCACGA
ace (adhesion of collagen)	ACE-F	GAATTGAGCAAAAGTTCAATCG
	ACE-R	GTCTGTCTTTTCACTTGTTTC
hdc (Histidine decarboxylase)	JV16HC	AGATGGTATTGTTTCTTATG
	JV17HC	AGACCATACACCATAACCTT
tdc (Tyrosine decarboxylase)	P2-for	GAYATNATNGGNATNGGNYTNGAYCARG
	P1-rev	CCRTARTCNGGNATAGCRAARTCNGTRTG
odc (Ornithine decarboxylase)	odc-3	GTNTTYAAYGCNGAYAARACNTAYTTYGT
	odc-16	ATNGARTTNAGTTCRCAYTTYTCNGG

Adopted from Eaton and Gasson (2001)[56]; and Casarotti et al (2017)[57]
